# Phenotypic Characterisation of the Abruzzo Donkey (*Equus asinus*), an Endangered Italian Genetic Resource: Body Measurements

**DOI:** 10.3390/ani16121932

**Published:** 2026-06-22

**Authors:** Ippolito De Amicis, Vincenzo Landi, Alberto De Berardinis, Medhat S. Saleh, Ivano Massirio, Domenico Robbe, Roberta Bucci, Augusto Carluccio

**Affiliations:** 1 Department of Veterinary Medicine, University of Teramo, Piano d’Accio, 64100 Teramo, Italy; ideamicis@unite.it (I.D.A.); alberto.deberardinis@unite.studenti.it (A.D.B.); imassirio@unite.it (I.M.); drobbe@unite.it (D.R.); acarluccio@unite.it (A.C.); 2 Department of Veterinary Medicine, University of Bari Aldo Moro, S.P. 62 per Casamassima km. 3, Valenzano, 70010 Bari, Italy; medhat.elshahat@uniba.it; 3 Department of Animal Production, Faculty of Agriculture, Benha University, 13736 Benha, Egypt; 4 Department of Clinical Sciences and Translational Medicine, University of Rome Tor Vergata, 00133 Rome, Italy; rbuccivet@gmail.com

**Keywords:** Abruzzo donkey, body size, dimensions, sexual dimorphism, biodiversity

## Abstract

The Abruzzo (AB) donkey is a mountain-adapted Italian population listed as a genetic resource at risk of extinction (census ≈ 600 animals; no studbook). We aimed to provide the first comprehensive morphometric description of the breed and to compare it with the Martina Franca (MF) donkey, its main progenitor. Sixty-nine adult donkeys (56 females, 13 males) from six farms were measured in 2024. Twenty-three linear traits plus body weight and body condition score were recorded three times by a single operator. Descriptive statistics, Welch’s *t*-test or Mann–Whitney U test with Benjamini–Hochberg correction, PCA and LDA with leave-one-out cross-validation were performed in R; comparison with MF was based on published summary statistics. Coefficients of variation for the three studbook-admission parameters were ≤0.10 in both sexes. Sixteen of 26 traits showed significant sex dimorphism, with the largest effect sizes for rump height, medial canthal distance and wither height. LDA correctly classified 94% of animals by sex. AB females were significantly smaller than MF in 22 of 23 shared traits but had a wider thorax (*p* = 0.012). The sexual dimorphism observed in the Abruzzo donkey is male-biased and predominantly size-based, with a minor and well-localised shape component in the head region. Males are significantly larger than females for all axial measurements (wither height A: +6.5 cm, +5.3%; rump height B: +6.4 cm, +5.0%; trunk length D: +12.1 cm, +9.5%), for thoracic circumference (M: +7.1 cm, +5.0%), for body weight (+49.9 kg, +20.6%) and for the main head traits (CM: +4.3 cm, +20.0%; G: +3.6 cm, +12.5%; H: +1.3 cm, +11.1%; E: +2.0 cm, +3.8%); no trait shows a significant female bias after BH-FDR correction. The AB donkey shows a uniform mesomorphic phenotype, smaller and stockier than MF, supporting the establishment of an official studbook.

## Simple Summary

The Italian endangered Abruzzo (AB) donkey population is a crucial genetic resource well-adapted to local environmental conditions. We measured 69 adult animals (56 females and 13 males) from six farms and compared them with published data on the Martina Franca (MF) donkey, the main progenitor of the breed. The three measurements that breeders’ associations use to enter a donkey into a studbook—height at withers, thoracic circumference and cannon circumference—showed low variability in both sexes, indicating that the AB donkey already has a recognisable phenotypic standard. Males were significantly larger than females for 16 of the 26 traits examined, and a multivariate model classified the sex of the animals with 94% accuracy from body measurements alone. Compared with the MF donkey, the AB donkey was smaller and lighter in all axial measurements but had a significantly wider thorax—a ‘smaller but stockier’ pattern consistent with adaptation to mountain transhumance. Our data provide the first comprehensive morphometric baseline of the AB donkey and support the proposal of establishing an official studbook to preserve this Italian genetic resource.

## 1. Introduction

Conservation of domestic animal genetic resources (AnGR) is recognised as a key component of global biodiversity and is explicitly promoted by the Convention on Biological Diversity and by the FAO through the Global Plan of Action for AnGR [1]. The IUCN Red List [2] and the FAO Domestic Animal Diversity Information System (DAD-IS) provide the reference framework to monitor the extinction risk of livestock breeds [3]. Donkeys (*Equus asinus*) have historically been used as draft and pack animals throughout the Mediterranean basin, but the mechanisation of agriculture has caused a dramatic decline in their numbers in Italy during the second half of the 20th century [4].

The classification of a livestock breed as ‘at risk of extinction’ is based on quantitative demographic criteria established at the international, European and national level. According to the FAO Domestic Animal Diversity Information System (DAD-IS), a breed is considered ‘endangered’ when its global population includes fewer than 100 breeding males or fewer than 1000 breeding females and the total population is declining [1]. At the European level, Council Regulation (EC) No. 1257/1999 introduced the concept of ‘local breeds in danger of being lost to farming’ and defined the eligibility thresholds for agro-environmental support. At the Italian level, Law 194/2015 (‘Disposizioni per la tutela e la valorizzazione della biodiversità di interesse agricolo e alimentare’) and the parallel regional implementations—for Abruzzo, the Deliberation of the Regional Council 1050/2018 establishing the Anagrafe Regionale dell’Agrobiodiversità—formalise the criteria for the inclusion of a local breed in the National and Regional Registries of Biodiversity, with the parallel designation of ‘Allevatori Custodi’. Applied to the Abruzzo donkey, all the cited criteria are met: the estimated total population is approximately 600 adult animals (with a strongly female-skewed sex ratio and only a very limited number of jacks maintained for reproduction), the breed is locally distributed and not yet supported by a formal studbook, and it is officially listed in the Anagrafe Regionale dell’Agrobiodiversità as a genetic resource at risk of extinction.

Italy officially recognises eight donkey breeds through genealogical studbooks—Amiata, Asinara, Martina Franca (MF), Pantesco, Ragusano, Romagnolo, Sardo and Viterbese. However, several local populations remain outside the studbook system despite a clear ethnological and cultural identity [5,6]. Among them, the Abruzzo (AB) donkey, a mountain-adapted population whose census is estimated at about 600 individuals, is currently registered by the Abruzzo Region as a genetic resource at risk of extinction [7]. Archival sources [8] indicate that the AB donkey derives from the crossing of autochthonous Apennine populations with MF stallions used at the Montereale and Foggia stations in the 1930s. Although regional archives provide basic information about the donkey herd in Abruzzo, these records do not constitute a formal system of pedigree or herd records. Therefore, detailed information about the pedigrees of the animals included in this study was not available. Efforts are currently underway, in cooperation with regional authorities and breeders’ associations, to establish a comprehensive system for registering this endangered genetic resource.

Abruzzo, a region between the central ridge of the Apennines and the Adriatic Sea, hosts three national parks and a regional park, representing one of the biodiversity hotspots of central Italy [9]. In this environment, donkeys have traditionally accompanied sheep flocks along the ‘tratturi’—grass tracks about 111 m wide used for transhumance between the mountains of L’Aquila and the Tavoliere plain of Apulia—and still play a role in agro-pastoral activities, agritourism, donkey-assisted interventions and milk production [10,11].

**Figure 1:**
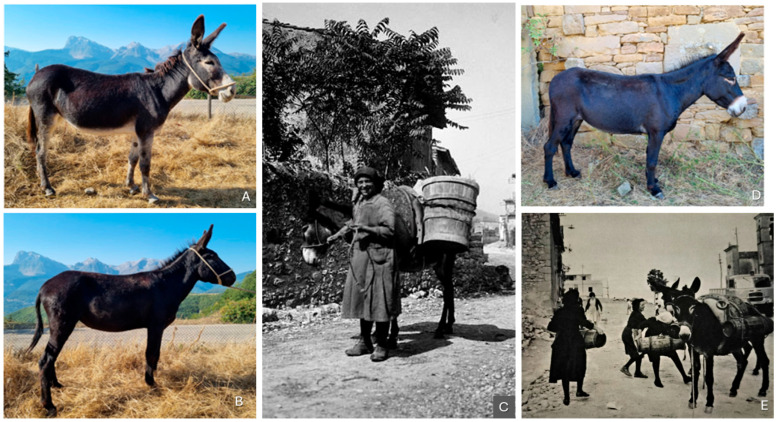
Phenotypic and traditional-use context of the Abruzzo donkey. (**A**) Adult Abruzzo donkey showing a dark-grey coat with light-grey muzzle and abdomen; (**B**) adult Abruzzo donkey showing a dark-bay coat with homogeneous dark muzzle and abdomen; (**D**) adult Abruzzo donkey showing a dark-bay coat with grey muzzle and light-grey markings on the abdomen—all three contemporary photographs were taken by the authors during the field measurement campaign and illustrate the typical coat variation in the breed. (**C**) Historical photograph of an Abruzzo donkey used as a means of transport of provender in Santa Rufina, L’Aquila (1929)—photo by Ugo De Pellis; (**E**) historical photograph documenting the transport of water by an Abruzzo donkey in Roio, L’Aquila (1962)—photo by Martelli. Panels (**C**,**E**) illustrate the traditional pack-and-transhumance use of the Abruzzo donkey in the Central Apennines.

The AB donkey is described as a mesomorphic equine of medium size, with the breed standard indicating a height at withers between 115 and 141 cm in males and 112 and 137 cm in females; thoracic circumference must be at least 100 cm in both sexes, and cannon circumference at least 19 cm in males and 17 cm in females (Figure 1) [7]. A recent study [12] characterised the phenotype of the MF donkey, but to the best of our knowledge no comprehensive morphometric description of the AB donkey has been published in scientific literature (Figure 1).

The classification of the Abruzzo donkey as a mesomorphic breed is supported here by quantitative criteria computed on the present sample. According to the classical zootechnical convention [13,14], an equine breed is classified as brachymorphic when the body index (BI = trunk length/thoracic circumference × 100) is below 86, as mesomorphic when BI is between 86 and 90, and as sub-doliquomorphic/doliquomorphic when BI exceeds 90. On the present sample, the body index averaged 90.5 ± 6.2 (range 75.5–100.8) in the whole population (89.9 ± 6.4 in females, 93.4 ± 4.5 in males); the thoracic index (L/N × 100) averaged 50.5 ± 5.3, the compactness index (Weight/D × 100) averaged 195.3 ± 41.3 kg/m of trunk length, and the gracility shin index (V/A × 100) averaged 14.2 ± 1.1. The female component of the breed falls clearly within the mesomorphic range, while the male component lies at the boundary between mesomorphic and sub-doliquomorphic, consistent with the known mild lengthening of the trunk in adult jacks. Taken together, these indexes support the classification of the Abruzzo donkey as a mesomorphic—with sub-doliquomorphic male tendency—equine breed of medium frame.

The current census of the Abruzzo donkey is estimated at approximately 600 adult animals, based on indirect figures derived from the National Livestock Database (Banca Dati Nazionale, BDN) and from the entries in the Anagrafe Regionale dell’Agrobiodiversità (Regione Abruzzo, DGR 1050/2018), since the breed is not yet officially recognised and no studbook or genealogical register is presently maintained. Direct count data are available only for the six farms enrolled in the present study, in which exhaustive census-level measurements were performed (*n* = 69 adult animals: 56 jennies and 13 jacks). Across the regional population the sex ratio is markedly female-skewed: the great majority of the breeding nucleus is constituted by adult jennies, while only a limited number of jacks are maintained for reproduction, as is typical of donkey breeding systems.

The aims of the present study were therefore: (i) to provide a quantitative description of the current AB donkey phenotype on the basis of 23 body measurements, body weight and BCS; (ii) to assess its sexual dimorphism through univariate and multivariate analyses; and (iii) to compare the AB donkey phenotype with that of the MF donkey, to inform future breed recognition and in situ conservation strategies.

## 2. Materials and Methods

### 2.1. Animals and Farms

**Figure 2:**
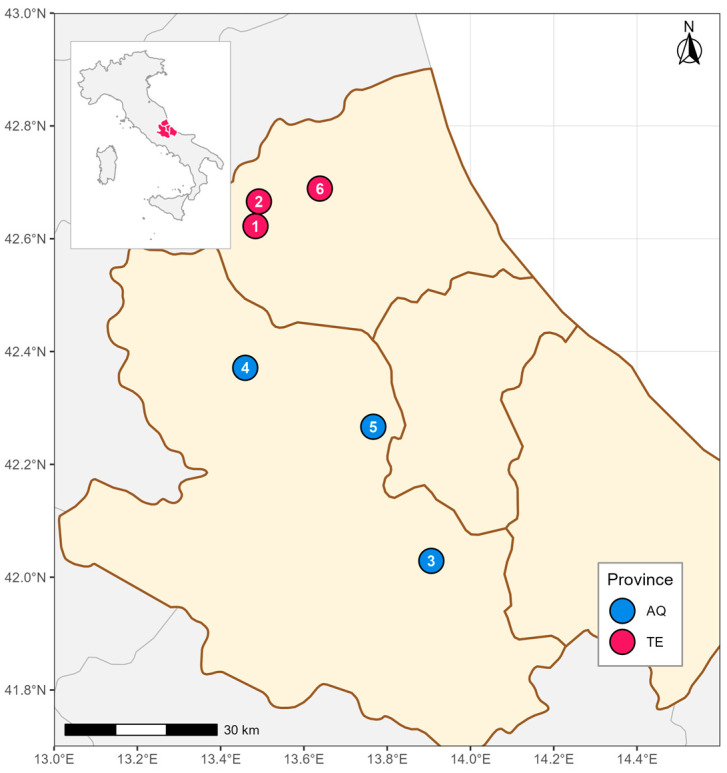
Geographical distribution of the six farms sampled within the Abruzzo region (Italy). In the Teramo province (TE): Crognaleto, Piano Roseto (1), Castagneto (2) and Rocca Santa Maria (6). In the L’Aquila province (AQ): Introdacqua (3), Tempera (4) and Capestrano (5).

A total of 69 adult AB donkeys (56 females, ≥3 yr; 13 males, ≥3 yr) from six farms located in the provinces of L’Aquila and Teramo (Abruzzo region, Italy) were measured between April and September 2024 (Figure 2). All animals were registered in the regional inventory of AB genetic resources [7]. Donkeys aged < 36 months, lame, visibly underweight (BCS < 2) or in late pregnancy (third trimester) were excluded from the study. The sample size corresponds to about 11% of the regional census of the breed.

The unbalanced sex ratio (56 females vs. 13 males) reflects the natural sex composition of the current Abruzzo donkey breeding population. In the absence of certified birth dates, dental examination was used as an inclusion criterion, and not as a continuous estimate of age, to ensure that all animals had completed somatic and skeletal maturity (≥3 years; permanent dentition fully erupted and lower corner incisors in wear). All animals retained in the analysis, therefore, belong to a single age stratum (mature adults within the productive window of the breed), which by design minimises the contribution of growth-related variability to the morphometric traits considered. This approach is the same as that adopted in the comparable phenotypic studies on registry-less donkey breeds [5,6,12,15] (e.g., Catalonian, Andalusian, Pega, and Martina Franca donkeys before the establishment of its studbook). All adult animals (≥3 years) available within each of the six farms at the time of the survey were measured, i.e., exhaustive census-level sampling of the accessible breeding nucleus, with juveniles excluded by the age criterion only. The age threshold of ≥3 years was adopted because by this age donkeys complete the eruption of the permanent incisors and most of the long-bone growth plate closure, and stabilise body weight when adequately nourished [16,17]; the same threshold was used in the recent characterisation of the Martina Franca donkey [12], ensuring direct comparability between the two studies. The per-farm distribution of the animals is reported in Supplementary File, Table S1, together with the geographic coordinates and the provincial assignment of each farm.

### 2.2. Body Measurements

Measurements were taken in compliance with Directive 2010/63/EU of the European Parliament and of the Council on the protection of animals used for scientific purposes [18]. Each animal was measured on a level, non-slippery surface, standing squarely with the head in neutral position, in the presence of a veterinarian. Measurements were taken three times by the same trained operator and averaged to reduce intra-observer variation; an inter-measurement difference > 1 cm triggered a fourth measurement. Instruments included a metric tape (Seca, Milan, Italy), a Hauptner–Herberholz hippometer (UnitedSportproducts Germany, GmbH, Birstein, Germany) and a digital animal scale (ABC Bilance, Modena, Italy), following the methodology applied to the MF donkey [12].

The 23 linear traits recorded were: height at withers (A), rump height (B), tail insertion height (C), trunk length (D), head length (E), distance between auditory meatuses (F), distance between temporal angles of the eyes (G), medial canthal distance (CM), intermandibular distance (H), ear length (I), chest width (L), thoracic circumference (M), thoracic height (N), thoracic width (O), rump length (P), anterior rump width (Q), posterior rump width (X), sternum-to-ground distance (R), shoulder length (S), knee-to-ground distance (T), knee circumference (U), cannon (shin) circumference (V), hock-to-ground distance (W) and hock circumference (Z). Body weight was recorded in kilograms. BCS was scored on the 1–5 donkey-specific scale [19]. Shoulder and rump angles could not be recorded reliably with the available equipment and were therefore omitted.

For the sake of reproducibility, H refers strictly to the intermandibular distance measured between the inner edges of the mandibular rami at the level of the mandibular notch, whereas CM refers to the distance between the medial angles of the two eyes.

### 2.3. Data Quality Control

The raw dataset was inspected for (i) duplicated records, (ii) univariate outliers (Tukey rule) and (iii) anatomical plausibility. Seven pairs of female records showing identical values in all 23 traits were identified as duplicate entries of the same field sheet and removed, leaving 56 unique females. One male showed an inversion between knee and hock measurements, corrected after cross-checking the original field sheet (Supplementary File; Table S2). BCS scores > 5 (*n* = 6) were treated as missing data because they were incompatible with the 1–5 donkey-specific scale.

### 2.4. Statistical Analysis

All analyses were performed in R v4.4 [20] using the tidyverse, rstatix, FactoMineR, factoextra, psych, MASS and effsize packages. Descriptive statistics (N, mean, standard deviation (SD), coefficient of variation (CV), median, interquartile range, minimum–maximum) were computed for the whole sample and by sex. Normality within sex was assessed with the Shapiro–Wilk test. Sexual dimorphism was tested with Welch’s *t*-test when both groups were normally distributed, and otherwise with the Mann–Whitney U test; *p*-values were adjusted with the Benjamini–Hochberg (BH) procedure to control the false discovery rate [21]. To assess the magnitude of sexual dimorphism, effect sizes were estimated as Cohen’s d (parametric) or Cliff’s δ (non-parametric) comparisons.

### 2.5. Zoometric Indexes: Ethnological and Functional Profile

The nine zoometric indexes computed on the present sample, separated into ethnological and functional categories, are reported in Supplementary File, Table S5. From the ethnological perspective, the body index (90.5 ± 6.2; 89.9 ± 6.4 in females, 93.4 ± 4.5 in males), the thoracic index (50.5 ± 5.3) and the cephalic index (34.1 ± 4.8) support the classification of the Abruzzo donkey as a mesomorphic equine breed, with a sub-doliquomorphic tendency in adult males. From the functional perspective, the gracility shin index (14.2 ± 1.1), the dactyl-thoracic index (12.3 ± 1.0), the compactness index (195.3 ± 41.3 kg/m of trunk), the weight-to-height ratio (2.02 ± 0.37 kg/cm), the pelvic relative width index and the proximodistal limb index jointly indicate an intermediate, light-to-medium functional profile, consistent with the historical use of the breed as a pack animal for transhumance and short-distance work in mountainous terrain rather than as a draft animal for heavy plain work. The morphological harmony of the breed, operationally quantified as the proportion of positive and significant (q < 0.05 BH-FDR) pair-wise correlations among the 25 linear traits, equals 53.0% (159/300 pairs) in the whole population and 52.0% (156/300) in females; both values are above the 50% threshold conventionally used to define a harmonic breed [13,14]. The much lower value observed in males (3.7%, 11/300) does not reflect a biological discordance but is a direct consequence of the very limited statistical power of the male sub-sample (*n* = 13), which is insufficient to reach significance under BH-FDR correction even for correlation coefficients of moderate-to-large magnitude.

Farm-of-origin effects on the morphometric profile were negligible: the mean intra-class correlation coefficient across the 26 quantitative traits was 0.031, and only three traits exceeded ICC = 0.15 (head width G, ICC = 0.226, *p* = 0.006; coxal-tuber width X, ICC = 0.157, *p* = 0.029; body condition score BCS, ICC = 0.187, *p* = 0.017). The multivariate PERMANOVA test on the standardised 22 linear traits did not detect any significant farm effect (vegan::adonis2, F = 0.92, *p* = 0.519, 999 permutations), indicating full overlap of the six farms in the multivariate phenotypic space (Figure S6). The significance of the sex effect on body size was preserved after adjustment for Farm in the two-way Type-III ANOVA (Supplementary File; Table S6): all axial traits (A, B, D, M), the head-size traits (CM, G), the limb traits (L, R, Z) and live weight retained significance at *p* < 0.05; head length (E), knee circumference (T) and BCS lost significance—consistent with their well-documented sensitivity to nutritional and environmental factors—while head width (H) and coxal-tuber width (X) gained significance, suggesting that the farm effect had previously masked these residual sex differences.

Within the classical framework of zootechnical phenotypic characterisation [13,14], the zoometric indexes computed on the present sample were organised into two categories. Ethnological indexes—describing the breed type—include the body index (BI = D/M × 100), the thoracic index (TI = L/N × 100) and the cephalic index (CeI = F/E × 100). Functional indexes—describing the productive aptitude of the breed—include the gracility shin index (GS = V/A × 100), the dactyl-thoracic index (DTI = V/M × 100), the compactness index (CoI = Weight/D × 100), the weight-to-height ratio (WHR = Weight/A), the pelvic relative width index (PRWI = Q/A × 100), and the proximodistal limb index (PDLI = (R − T)/R × 100). The morphological harmony of the breed was operationally quantified, as classically done in equine ethnology, as the proportion of pair-wise Pearson correlations among the linear measurements that are positive and significant at Benjamini–Hochberg-adjusted q < 0.05; a breed is considered harmonic when this proportion exceeds 50% [15,22]. Comparison with the Martina Franca donkey was performed at the population-mean level on the published summary statistics [12]; the individual-level MF dataset is not publicly available, which precludes the joint multivariable modelling of age, herd, year, season and operator as covariates. To minimise this limitation, the present study adopted the same set of 23 linear traits, the same instruments, the same triplicate-measurement protocol, the same age criterion (≥3 years), and the same body-condition inclusion rule as in the MF study.

To assess whether the farm of origin acted as a confounder of the breed-level phenotypic description, the variance of each linear morphometric trait was partitioned between farm and within-farm residual components using a one-way random-effects model (intra-class correlation coefficient, ICC; [23]) on the female subset (*n* = 56). The multivariate effect of farm on the morphometric profile was tested by permutational multivariate analysis of variance (PERMANOVA, [24]; 999 permutations) on the Euclidean distance matrix of the standardised 22 linear traits. To verify that sexual-dimorphism results were not driven by an unbalanced sex/farm distribution, each Welch’s *t*-test was paralleled by a two-way fixed-effects ANOVA of the form trait ~ Sex + Farm and the partial Type-III F-test for the sex effect was compared with the corresponding univariate *p*-value. Results are reported in Supplementary File, Tables S5 and S6 and Figure S6.

Pairwise Pearson correlations were computed with BH-corrected *p*-values. Principal component analysis (PCA) on the correlation matrix was performed on the complete-case dataset (BCS excluded). Linear discriminant analysis (LDA) with leave-one-out cross-validation was used to quantify the separation between sexes on the basis of morphometric traits. Classical zoometric indices (body index = D/M × 100; thoracic index = L/N × 100; dactyl-thoracic index = V/M × 100; gracility index = V/A × 100; weight-for-height = weight/A) were computed. Comparison with the MF donkey was performed on published summary statistics [12] using Welch’s *t*-tests on means, SDs and sample sizes, with BH-FDR correction. Significance was set at α = 0.05.

## 3. Results

**Figure 3:**
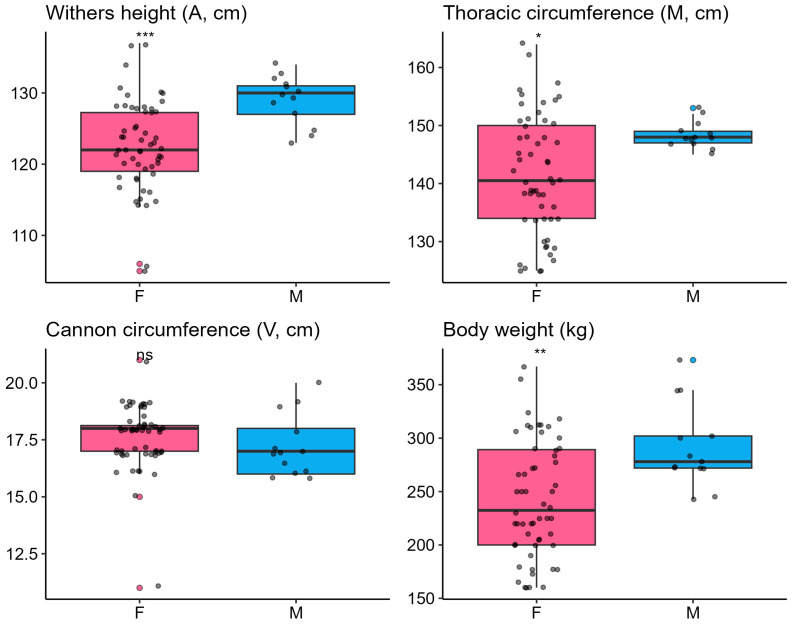
Boxplots of the three studbook-admission parameters (A, M, V) and body weight by sex (F, females; M, males). Each dot represents an individual animal; significance levels refer to Wilcoxon tests, * *p* < 0.05, ** *p* < 0.01, *** *p* < 0.001; ns, not significant.

**Figure 4:**
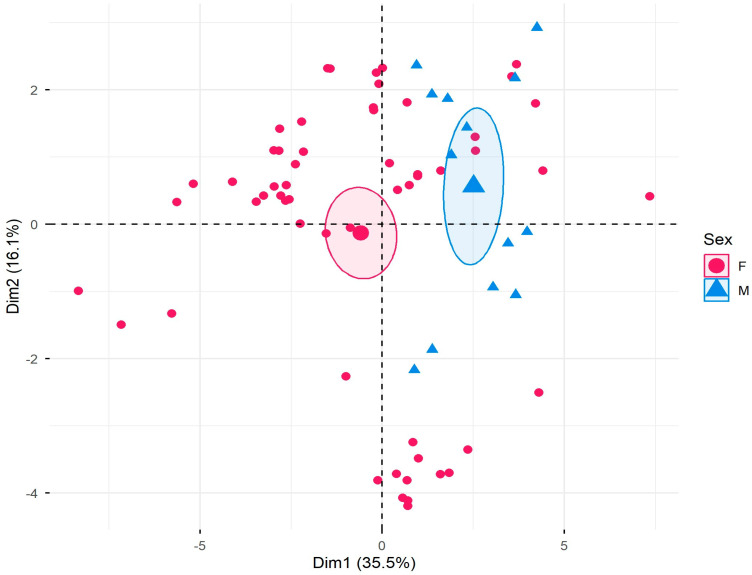
PCA individual plot (Dim1 = 35.5%, Dim2 = 16.1%) coloured by sex with 95% confidence ellipses. Males (blue) are displaced towards larger sizes along Dim1.

**Figure 5:**
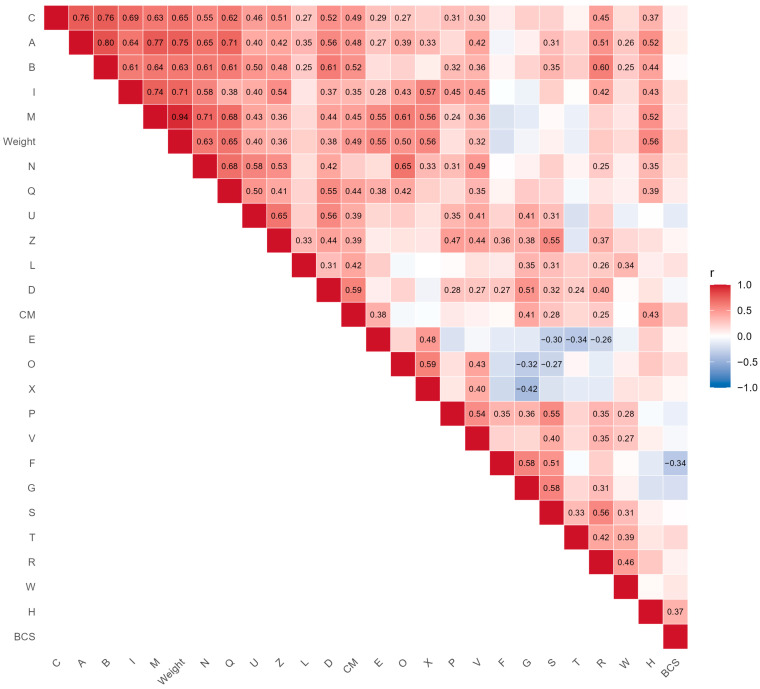
Heat-map of Pearson correlations among morphometric traits in the whole population; only correlations significant at BH-adjusted *p* < 0.05 are shown.

**Figure 6:**
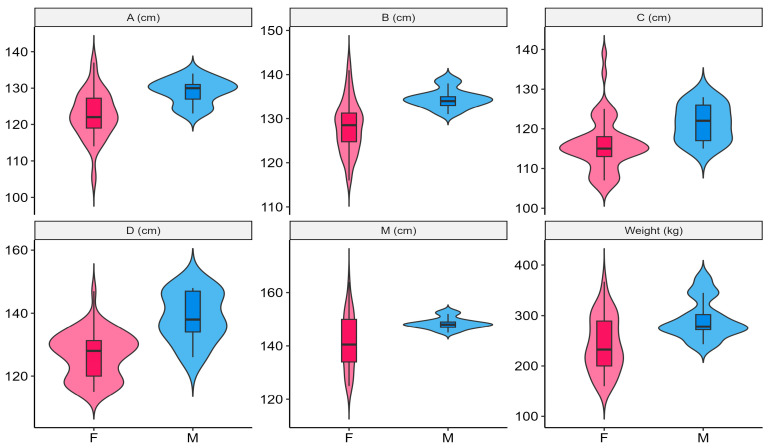
Violin plots of axial measurements (A, B, C, D), thoracic circumference (M) and body weight by sex (F, females; M, males).

Descriptive statistics are reported by sex in Table 1 and are visualised in Figure 3, Figure 4, Figure 5 and Figure 6 for the most relevant traits. Sexual dimorphism is summarised in Table 2 and the comparison with Martina Franca donkey in Table 3.

### 3.1. Descriptive Morphometry

Descriptive statistics for females and males are reported in Table 1. In females, wither height averaged 122.6 ± 6.4 cm (range 105–137), thoracic circumference 141.4 ± 10.3 cm (125–164) and cannon circumference 17.6 ± 1.4 cm (11–21); mean body weight was 242.5 ± 54.3 kg. In males, the corresponding values were 129.1 ± 3.4 cm, 148.5 ± 2.2 cm and 17.3 ± 1.3 cm, with a mean body weight of 292.4 ± 39.5 kg. CV values for the three studbook-admission parameters (A, M, V) were ≤0.10 in both sexes, while the highest CV values were observed for thoracic width (O, 0.14–0.20), posterior rump width (X, 0.18–0.19) and body weight (0.14–0.22). Figure 3 illustrates the distribution of the three studbook-admission parameters and body weight by sex.

### 3.2. Sexual Dimorphism

Males were significantly larger than females in 16 of the 26 traits tested after BH-FDR correction (Table 2 and Supplementary File; Table S4). The largest effect sizes were observed for rump height (B, Cohen’s d = −1.16), medial canthal distance (CM, Cliff’s δ = −0.94), wither height (A, d = −1.08), body weight (d = −0.96) and thoracic circumference (M, d = −0.75). Posterior rump width (X), although numerically wider in females, did not survive multiple-testing correction. Dimorphism was therefore predominantly driven by axial size and head traits rather than by pelvic conformation in our sample.

**Table 1:** Morphometric measurements of females (*n* = 56) and males (*n* = 13) of the Abruzzo donkeys. A, height at withers; B, rump height; C, tail-insertion height; D, trunk length; E, head length; F, distance between auditory meatuses; G, distance between temporal angles of the eyes; CM, medial canthal distance; H, intermandibular distance; I, ear length; L, chest width; M, thoracic circumference; N, thoracic height; O, thoracic width; P, rump length; Q, anterior rump width; X, posterior rump width; R, sternum-to-ground distance; S, shoulder length; T, knee-to-ground distance; U, knee circumference; V, cannon (shin) circumference; W, hock-to-ground distance; Z, hock circumference; Weight, body weight (kg); BCS, body condition score (1–5 donkey-specific scale). All linear measurements are expressed in centimetres unless otherwise stated. SD; standard deviation; CV, coefficient variation; Min–Max, minimum–maximum.

Trait (cm)	Females				Males			
	Mean ± SD	CV	Min–Max	Median	Mean ± SD	CV	Min–Max	Median
A	122.62 ± 6.37	0.05	105–137	122	129.08 ± 3.43	0.03	123–134	130
B	128.16 ± 5.97	0.05	116–143	128.5	134.54 ± 2.22	0.02	131–139	134
C	115.86 ± 6.51	0.06	105–139	115	121.46 ± 4.58	0.04	115–128	122
D	126.61 ± 7.21	0.06	115–147	128	138.69 ± 7.73	0.06	126–148	138
E	53.96 ± 4.75	0.09	47–66	52	56 ± 1.58	0.03	53–58	56
F	18.55 ± 2.07	0.11	14–22	18	17.62 ± 1.33	0.08	15–19	18
G	28.52 ± 3.8	0.13	18–36	29.5	32.08 ± 2.5	0.08	27–36	32
CM	21.41 ± 1.4	0.07	19–24	22	25.69 ± 1.38	0.05	22–27	26
H	11.21 ± 1.29	0.11	9–14	11	12.46 ± 2.02	0.16	9–15	13
I	32.2 ± 2.38	0.07	28–37	32	32.77 ± 1.69	0.05	30–36	33
L	28.29 ± 2.42	0.09	19–33	29	30.77 ± 1.88	0.06	28–34	30
M	141.41 ± 10.3	0.07	125–164	140.5	148.46 ± 2.22	0.01	145–153	148
N	56.89 ± 4.17	0.07	46–67	57	58.54 ± 2.88	0.05	55–64	58
O	47.89 ± 6.56	0.14	23–61	48	45.46 ± 9.23	0.2	29–57	45
P	42.8 ± 2.54	0.06	38–49	42	42 ± 2.04	0.05	39–46	42
Q	40.54 ± 2.97	0.07	35–54	40.5	44 ± 2.12	0.05	40–47	44
X	26.5 ± 4.76	0.18	20–37	25	24.31 ± 4.66	0.19	19–33	24
R	67.07 ± 4.22	0.06	57–75	67	69.69 ± 3.57	0.05	60–73	71
S	45.04 ± 3.06	0.07	39–50	45	46.65 ± 4.01	0.09	40–51	48
T	39.45 ± 2.62	0.07	27–44	39.5	40.65 ± 1.41	0.03	39–43	41
U	27.36 ± 2.9	0.11	23–44	27	29.38 ± 2.69	0.09	26–35	29
V	17.62 ± 1.39	0.08	11–21	18	17.27 ± 1.33	0.08	16–20	17
W	48.32 ± 5.01	0.1	28–56	49	47.77 ± 5.12	0.11	35–53	50
Z	34.38 ± 3.49	0.1	29–49	34	36.62 ± 3.38	0.09	27–41	37
Weight (kg)	242.5 ± 54.26	0.22	160–367	232.5	292.38 ± 39.5	0.14	243–373	278
BCS	3.62 ± 0.64	0.18	2–5	4	4.33 ± 0.87	0.2	3–5	5

The sexual dimorphism observed in the Abruzzo donkey is male-biased and predominantly size-based, with a minor and well-localised shape component in the head region. Males are significantly larger than females for all axial measurements (wither height A: +6.5 cm, +5.3%; rump height B: +6.4 cm, +5.0%; trunk length D: +12.1 cm, +9.5%), for thoracic circumference (M: +7.1 cm, +5.0%), for body weight (+49.9 kg, +20.6%) and for the main head traits (CM: +4.3 cm, +20.0%; G: +3.6 cm, +12.5%; H: +1.3 cm, +11.1%; E: +2.0 cm, +3.8%); no trait shows a significant female bias after BH-FDR correction. Size dimorphism is supported by the very large effect sizes for size-related traits (Cohen’s d up to −1.16 for rump height; Cliff’s delta up to −0.94 for medial canthal distance), by the dominance of PC1 in the principal component analysis (35.1% of the total variance) loaded by all axial and girth traits, and by the displacement of male individuals along PC1 in the PCA plot. A more limited but unambiguous shape component is observed in the head region: the male/female ratios of head traits (CM: +20.0%, G: +12.5%, H: +11.1%) are clearly higher than those of axial traits (A: +5.3%, B: +5.0%, M: +5.0%), indicating a disproportionate enlargement of the head relative to body size in males.

**Table 2:** Sexual dimorphism in Abruzzo donkeys: traits with a significant difference between females (*n* = 56) and males (*n* = 13) after Benjamini–Hochberg correction. Effect sizes expressed as Cohen’s d (Welch’s *t*-test) or Cliff’s δ (Mann–Whitney test). Negative values indicate F < M.

Trait	Test	Mean (Female)	Mean (Male)	Diff	*p*-Value	Effect Size Type	Effect Size	q-Value
B	Welch’s *t*-test	128.16	134.54	6.38	<0.000001	Cohen’s d	−1.16	0.00000112
CM	Mann–Whitney	21.41	25.69	4.28	<0.000001	Cliff’s delta	−0.94	0.00000112
A	Welch’s *t*-test	122.62	129.08	6.45	0.0000144	Cohen’s d	−1.08	0.0000955
M	Welch’s *t*-test	141.41	148.46	7.05	0.0000147	Cohen’s d	−0.75	0.0000955
D	Mann–Whitney	126.61	138.69	12.09	0.0000366	Cliff’s delta	−0.74	0.000190
Q	Mann–Whitney	40.54	44	3.46	0.0000563	Cliff’s delta	−0.72	0.000244
G	Mann–Whitney	28.52	32.08	3.56	0.000395	Cliff’s delta	−0.63	0.001466
L	Mann–Whitney	28.29	30.77	2.48	0.000674	Cliff’s delta	−0.6	0.002192
C	Mann–Whitney	115.86	121.46	5.6	0.000963	Cliff’s delta	−0.59	0.002504
Weight (kg)	Welch’s *t*-test	242.5	292.38	49.88	0.000888	Cohen’s d	−0.96	0.002504
Z	Mann–Whitney	34.38	36.62	2.24	0.004779	Cliff’s delta	−0.5	0.011296
U	Mann–Whitney	27.36	29.38	2.03	0.005797	Cliff’s delta	−0.49	0.012561
H	Mann–Whitney	11.21	12.46	1.25	0.015435	Cliff’s delta	−0.43	0.029510
BCS	Mann–Whitney	3.62	4.33	0.71	0.015890	Cliff’s delta	−0.47	0.029510
E	Mann–Whitney	53.96	56	2.04	0.021733	Cliff’s delta	−0.41	0.037671
R	Mann–Whitney	67.07	69.69	2.62	0.028571	Cliff’s delta	−0.39	0.046428
T	Mann–Whitney	39.45	40.65	1.21	0.077280	Cliff’s delta	−0.31	0.118193
F	Mann–Whitney	18.55	17.62	−0.94	0.086565	Cliff’s delta	0.3	0.118513
S	Mann–Whitney	45.04	46.65	1.62	0.086606	Cliff’s delta	−0.31	0.118513
N	Mann–Whitney	56.89	58.54	1.65	0.113053	Cliff’s delta	−0.28	0.146969
X	Mann–Whitney	26.5	24.31	−2.19	0.119636	Cliff’s delta	0.28	0.148121
V	Mann–Whitney	17.62	17.27	−0.36	0.145902	Cliff’s delta	0.25	0.172429
P	Welch’s *t*-test	42.8	42	−0.8	0.236617	Cohen’s d	0.33	0.267481
I	Welch’s *t*-test	32.2	32.77	0.57	0.322258	Cohen’s d	−0.25	0.349113
O	Mann–Whitney	47.89	45.46	−2.43	0.424069	Cliff’s delta	0.14	0.441032
W	Mann–Whitney	48.32	47.77	−0.55	0.913876	Cliff’s delta	0.02	0.913876

For trait abbreviations, see Table 1.

### 3.3. Multivariate Structure

The first two principal components explained 51.6% of the total variance (PC1 = 35.5%, PC2 = 16.1%). PC1 was dominated by positive loadings of size-related traits (A, B, C, D, I, M, N, Z, weight), while PC2 opposed limb and proportional traits (T, F, G, S) to head length and posterior rump width (E, H, X). The 95% confidence ellipses for the two sexes overlapped but showed a clear shift along PC1, with males displaced towards larger sizes (Figure 4). Linear discriminant analysis with leave-one-out cross-validation correctly classified 93.7% of the animals by sex (91% of females, 100% of males), confirming the overall strength of sexual dimorphism when considered multivariate (Supplementary File; Figures S1 and S2).

### 3.4. Correlation Structure

Pairwise correlations were generally positive (Figure 5). After BH-FDR correction, correlations involving height traits (A, B, C) and thoracic traits (M, N, O) formed a tight cluster with r > 0.5. Body weight was correlated with M, A, B and D at r > 0.6 in both sexes (Supplementary File; Figures S3 and S4). Violin plots of the main axial measurements and body weight by sex are shown in Figure 6 (Supplementary File; Figure S5). Quantitative analysis of the pair-wise Pearson correlations among the 25 linear morphometric traits (*n* = 300 correlations) supports the morphological harmony of the Abruzzo donkey: 159/300 correlations (53.0%) are positive and significant after Benjamini–Hochberg correction in the whole population, and 156/300 (52.0%) in the female component, both exceeding the conventional 50% zootechnical threshold [13,14,19]). In the male sub-sample, only 11/300 (3.7%) survive the BH correction; this reflects the limited statistical power of the male sample (*n* = 13) rather than a biological discordance, as discussed in the limitations of this study. The Abruzzo donkey can therefore be confidently described as a harmonic breed, with a fully harmonic conformation in the females (Table S7).

### 3.5. Zoometric Indices

**Figure 7:**
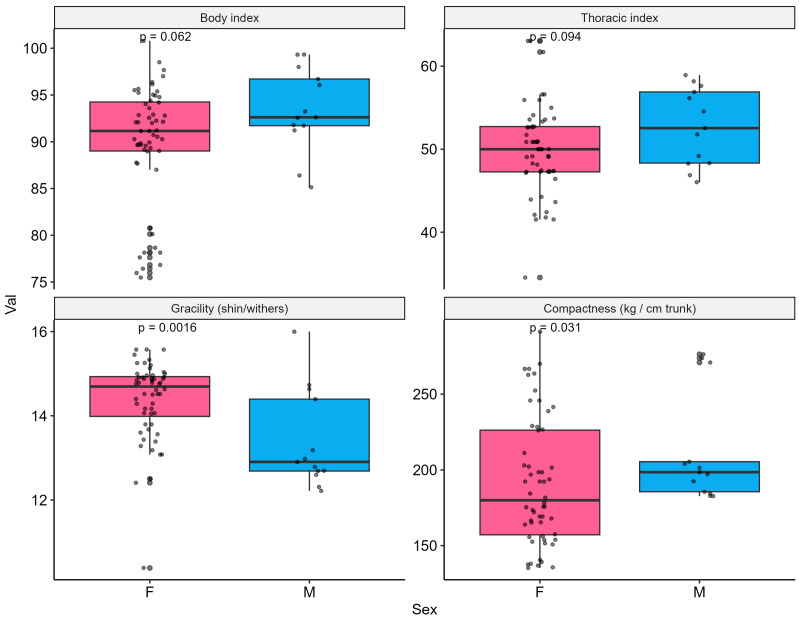
Zoometric indices (body, thoracic, gracility, compactness) by sex. *p*-values refer to Wilcoxon tests. Each dot represents an individual animal.

The zoometric indices computed on the AB donkeys are presented in Figure 6 and Figure 7. Body index, thoracic index, gracility index and compactness index were comparable between sexes, except for the compactness index, which was slightly higher in males.

### 3.6. Comparison with the Martina Franca Donkey

Compared with MF females (*n* = 73 [12]), AB females were significantly smaller for 22 of the 23 shared traits (Table 3). All axial traits (A: 122.6 vs. 137.6; B: 128.2 vs. 141.4; C: 115.9 vs. 129.4; D: 126.6 vs. 143.8), the main girths (M: 141.4 vs. 159.6; V: 17.6 vs. 19.1), body weight (242.5 vs. 331.4 kg, −27%) and most head and limb traits were reduced in AB (all q < 0.001). A notable exception was thoracic width (O), which was significantly larger in AB females (47.9 vs. 45.1 cm, q = 0.012), suggesting a more compact thoracic conformation in the AB breed.

**Table 3:** Comparison between female Abruzzo (AB, *n* = 56) and Martina Franca (MF, *n* = 73 [12]) donkeys (Welch’s *t*-tests on summary statistics, with Benjamini–Hochberg correction). Thoracic width (O) is the only trait with a reversed direction (AB > MF).

Trait	Mean AB	SD AB	N AB	N MF	Mean MF	SD MF	t	df	*p*-Value	q-Value
T	39.45	2.62	56	73	41.14	2.18	3.91	106	0.000164	0.000197
P	42.8	2.54	56	73	44.35	2	3.75	102.1	0.000293	0.000335
W	48.32	5.01	56	73	50.83	3.4	3.22	92	0.001766	0.001926
X	26.5	4.76	56	73	28.5	3.18	2.72	91	0.007917	0.008261
O	47.89	6.56	56	73	45.1	5.65	−2.54	108.6	0.012345	0.012345
A	122.62	6.37	56	73	137.6	4.78	14.69	98.7	<0.000001	<0.000001
B	128.16	5.97	56	73	141.4	4.73	13.64	102.6	<0.000001	<0.000001
C	115.86	6.51	56	73	129.4	4.46	13.35	92.6	<0.000001	<0.000001
D	126.61	7.21	56	73	143.8	5.54	14.8	100.4	<0.000001	<0.000001
E	53.96	4.75	56	73	60.06	3.16	8.3	90.7	<0.000001	<0.000001
F	18.55	2.07	56	73	20.7	1.44	6.62	93.5	<0.000001	<0.000001
G	28.52	3.8	56	73	34.29	1.88	10.42	75.5	<0.000001	<0.000001
H	11.21	1.29	56	73	24.81	1.13	62.6	109.8	<0.000001	<0.000001
I	32.2	2.38	56	73	36.4	1.7	11.19	95.2	<0.000001	<0.000001
L	28.29	2.42	56	73	31.97	2.14	9.01	110.5	<0.000001	<0.000001
M	141.41	10.3	56	73	159.6	6.65	11.5	88.9	<0.000001	<0.000001
N	56.89	4.17	56	73	63.87	3.76	9.82	111.7	<0.000001	<0.000001
Q	40.54	2.97	56	73	46.32	2.56	11.64	108.7	<0.000001	<0.000001
R	67.07	4.22	56	73	71.03	3.98	5.41	114.8	<0.000001	<0.000001
S	45.04	3.06	56	73	49.23	2.67	8.16	109.6	<0.000001	<0.000001
U	27.36	2.9	56	73	29.95	1.72	5.94	84	<0.000001	<0.000001
V	17.62	1.39	56	73	19.08	1.16	6.32	106.2	<0.000001	<0.000001
Z	34.38	3.49	56	73	38.84	2.43	8.18	93.7	<0.000001	<0.000001
Weight (kg)	242.5	54.26	56	73	331.4	37.3	10.5	92.8	<0.000001	<0.000001

N, number of animals; SD; standard deviation; df, degree of freedom.

## 4. Discussion

Our results provide the first comprehensive morphometric baseline of the AB donkey and confirm that the breed is a morphologically uniform population of mesomorphic type, clearly distinct from the Martina Franca donkey. The consistently low coefficients of variation observed for the three studbook-admission parameters (A, M, V) indicate that decades of informal selection by local breeders have produced a recognisable standard, despite the absence of a formal breed association. The mean values for wither height (122.6 cm in females and 129.1 cm in males) and for the thoracic and cannon circumferences fall within the breed standard proposed by the Abruzzo regional inventory [7], supporting the phenotypic consistency of the population.

The ethnological profile of the breed, supported by the body index, thoracic index and cephalic index, places the Abruzzo donkey within the mesomorphic spectrum, with a mild sub-doliquomorphic tendency in adult males. The functional profile, supported by the gracility shin, dactyl-thoracic, compactness and weight-to-height ratio indexes, is intermediate and points to a pack/short-distance aptitude rather than to a heavy draft aptitude, in agreement with the historical use of the breed in the transhumance pathways of the central Apennines. The morphological harmony of the breed, classically quantified as the proportion of positive and significant pair-wise correlations among the linear measurements, reaches 53.0% in the whole population and 52.0% in females, both above the 50% conventional threshold of harmonic conformation; the apparent low value observed in the male sub-sample (3.7%) does not reflect a biological discordance but a well-known limitation of statistical power under BH-FDR correction in small samples.

The dedicated analysis of the farm-of-origin effect (ICC, PERMANOVA, two-way ANOVA—Tables S5 and S6 and Figure S6) demonstrates that, despite the unbalanced distribution of animals across the six farms (Farms A + B accounting for 71% of the sample), the morphological profile of the Abruzzo donkey is fully homogeneous across farms. The multivariate PERMANOVA detected no significant farm clustering (vegan::adonis2 F = 0.92, *p* = 0.519), the mean ICC was 0.031, and the conclusions on sexual dimorphism were preserved after correction for farm. The only two traits with a non-negligible farm effect—body condition score (ICC = 0.19) and head width (ICC = 0.23)—are well-known in the donkey and horse literature to be sensitive to management and feeding plans (BCS) and to display residual ontogenetic variability at maturity (cranial width). This result supports the interpretation of the values reported in Table 1, Table 2 and Table 3 as genuine breed-level descriptors rather than as the expression of two dominant management environments and strengthens the proposed phenotypic standard of the Abruzzo donkey.

The magnitude of sexual dimorphism observed in AB is of the expected direction for a mesomorphic donkey [12,22]: males were heavier and taller, with more pronounced head traits. Interestingly, contrary to a priori expectations, posterior rump width (X) did not show a statistically significant sex effect in our sample after BH correction. This finding, together with the very high cross-validated accuracy of the LDA model (93.7%) (Supplementary File; Table S3), indicates that sex can be reliably predicted from body measurements even in the absence of a classical pelvic dimorphism, and that head-related traits (CM and H) carry substantial discriminant information. Similar dimorphic patterns have been described in the Catalonian donkey [15,22] and in other Mediterranean donkey populations [25].

The comparison with MF donkeys reveals a systematic reduction in size and weight of AB animals, in the range of −10% to −30% depending on the trait, with the sole exception of thoracic width. This ‘smaller but wider thorax’ pattern is consistent with the history of the two breeds: while MF donkeys were selected for heavy draft and mule production in the Apulian plain, AB donkeys adapted to the steep terrain of the central Apennines and to transhumance, where a shorter, stockier body conformation is biomechanically advantageous for negotiating uneven ground and carrying concentrated loads on short distances. The preservation of this unique thoracic-dominant conformation should be considered a priority within the Abruzzo regional plan for the conservation of AnGR, aligned with the principles of the Convention on Biological Diversity [26] and with the methodological framework proposed for assessing the cultural value of local livestock breeds [27]. The increasing interest in the breed for agritourism and educational farms further underscores its cultural and economic significance [28,29].

The main limitations of this study are the unbalanced sex ratio (56 F vs. 13 M), reflecting the natural composition of the breeding population, the lack of age and pedigree data, and the absence of shoulder and rump angle measurements. Although this reflects the current demographic composition of the population, the small sample size of males may have reduced the statistical ability to detect sex-related differences and may have affected the observed variance in graphical comparisons. Therefore, the results of sexual-based comparisons should be interpreted with caution. Moreover, the comparison with MF is based on summary statistics from a previously published study [12] rather than on individual-level data, which precludes a joint multivariate analysis of the two breeds. Another potential limitation of this study is that the morphometric data for the Martina Franca population were obtained from a previously published dataset and were not collected by the same operator who measured the Abruzzo donkeys. Therefore, the possibility of discrepancies between observers cannot be ruled out, and this must be taken into account when interpreting comparative results. Future work should address these limitations by combining individual-level datasets of both breeds and by integrating molecular markers (SNP panels) [6,30] to disentangle the relative contribution of genetic drift, founder effect and artificial selection to the phenotypic divergence documented here.

The unbalanced sex ratio of our sample (56 females, 13 males) reflects the natural demographic structure of the breed, in which only a limited number of jacks are kept for reproduction, rather than a sampling choice. All adult males available in the six farms were measured; any further increase in the male sample would require enrolling additional farms outside the currently surveyed area, the existence of which is not yet documented and is one of the explicit goals of the future regional registry. Because the analysed dataset corresponds to the exhaustive census of the six farms, classical mixed models including farm as a random effect would have been conceptually and statistically inappropriate; we therefore acknowledge that the between-farm component of variance cannot be formally partitioned with the present data. The comparison with the Martina Franca donkey is based on previously published summary statistics [12] rather than on individual-level data, which precludes a joint mixed-model analysis adjusted for potential confounders (operator, herd, year, season, age). To minimise this limitation, the AB protocol was harmonised a priori with the MF protocol with respect to traits, instruments, operator-within-study design, age threshold and season of data collection. Age could potentially be estimated by dental ageing [31] in future surveys of the breed; this approach was not adopted in the present study because all animals were classified as adults (≥3 years) by the on-site veterinarian, which was the only age stratification needed for the inclusion criterion. Animal age was assessed in vivo by dental examination on each animal (eruption pattern of the permanent incisors, presence and degree of wear of the lower corner incisors, dental tables and infundibulum morphology). Because the Abruzzo donkey currently lacks an official studbook and no birth records were available for the surveyed animals, the dental examination was used as an inclusion criterion only, and not as a continuous estimate of age, to ensure that all enrolled subjects had completed somatic and skeletal maturity (≥3 years). All animals included in the analysis therefore belong to a single age stratum (mature adults within the productive window of the breed), which minimises by design the contribution of growth-related variability to the linear morphometric traits. The very high cross-validated accuracy of the LDA model (93.7%) indicates that, despite the limited male sample size, the morphometric signal of the male group is robust. Comparisons between the donkey populations of AB and MF should be interpreted with caution. Although both populations were assessed using similar morphological measurement methodologies, many factors that could influence body measurements were not explicitly controlled for in this study, including age, herd, management and dietary conditions, year of measurement, and potential operator-related variability. Previous studies on donkeys have shown the effect of age and sex on morphometric traits, while the environmental and management conditions of each population may contribute more to phenotypic variation [12,15,32]. Therefore, observed differences should be considered descriptive variations at the group level, and not merely characteristics associated with the breed. Future studies that rely on larger and more balanced datasets and incorporate these sources of variance within a unified statistical framework, would allow for a more accurate assessment of differences between populations.

## 5. Conclusions

The AB donkey shows a recognisable and uniform mesomorphic phenotype, smaller and lighter than the Martina Franca progenitor but more compact in the thoracic region, with significant sexual dimorphism reliably captured by linear discriminant analysis. Our data represents the first comprehensive morphometric baseline of the AB donkey and provides a robust basis for the establishment of an official studbook, for in situ conservation and for the socio-economic valorisation of the breed within agrotourism and ethical donkey-assisted interventions.

Quantitative analysis of the zoometric indexes confirms the ethnological classification of the Abruzzo donkey as a mesomorphic breed (body index ≈ 90, mesomorphic in females and at the mesomorphic/sub-doliquomorphic boundary in males) and the functional profile indicates an intermediate, pack-and-transhumance functional aptitude. Furthermore, the morphological harmony of the breed exceeds the 50% conventional threshold both in the whole population (53.0%) and in the female component (52.0%). These results support the proposed phenotypic standard of the Abruzzo donkey and provide a quantitative baseline for the future establishment of an official studbook.

## Data Availability

The original contributions presented in this study are included in the article and the Supplementary Materials. The raw dataset and the R analysis script will be available at the corresponding author.

## Supplementary Materials

The following supporting information can be downloaded at https://www.mdpi.com/article/10.3390/ani16121932/s1, Figure S1: Principal component analysis (PCA): scree plot showing the percentage of total variance explained by each principal component (PC1 to PC25). PC1 captures the dominant size-related variation; PC2 captures the main proportional/conformation gradient. The ‘elbow’ criterion suggests that the first two components are sufficient to summarise the morphometric variability in the dataset; Figure S2: PCA variable contribution plot. Each arrow represents one of the 25 morphometric traits projected on the plane of the first two principal components (PC1 vs. PC2). Arrow length reflects the trait’s loading on the two axes and arrows are coloured by their relative contribution to the explained variance (cool to warm gradient). Variables pointing in the same direction are positively correlated; opposite directions indicate negative correlation; Figure S3: Pearson correlation heat-map of the 25 morphometric traits in female Abruzzo donkeys (*n* = 56). Only correlations statistically significant after Benjamini–Hochberg correction (q < 0.05) are displayed. Cell colour intensity reflects the magnitude and sign of the correlation coefficient (blue = positive, red = negative). Hierarchical clustering of traits was used to organise the matrix; Figure S4: Pearson correlation heat-map of the 25 morphometric traits in male Abruzzo donkeys (*n* = 13). Display rules and clustering are as in Figure S3. The lower number of significant correlations compared to females reflects the smaller sample size of the male group and should not be interpreted as biological discordance; Figure S5: Kernel density plot of body weight (kg) distributions by sex in the Abruzzo donkey sample. Females (pink) and males (blue) are shown on the same scale, with each curve normalised to integrate to one. The horizontal shift highlights the sexual dimorphism in body mass (mean F = 242.5 kg; mean M = 292.4 kg); Figure S6: Principal Component Analysis (PCA) of the 22 linear morphometric traits in the 56 adult females of the Abruzzo donkey, colored by farm of origin (Farms A–F, see Table S5). Confidence ellipses (95%) of farms with *n* ≥ 3 individuals overlap entirely, indicating the absence of multivariate farm clustering (PERMANOVA F = 0.92, *p* = 0.519, 999 permutations; vegan::adonis2). This result, together with the negligible average intra-class correlation across single traits (mean ICC = 0.031; only the head width G, the coxal-tuber width X and the body condition score BCS—environmentally and ontogenetically labile traits—exceed ICC = 0.15), supports the morphological homogeneity of the breed across the surveyed farms. The PCA plot is produced by the analisi_asino_abruzzese_v5.R script; Table S1: Distribution of the *n* = 69 Abruzzo donkeys among the six farms enrolled in the present study. Farms are anonymized (Farm A–F) to protect the privacy of the owners; only municipality and province are reported. All adult animals (≥3 years) present in each farm at the time of the survey were measured (exhaustive census-level sampling at farm level); Table S2: Cleaned full dataset of Abruzzo donkey morphometric measurements (*n* = 69: 56 females + 13 males) used in the statistical analyses, after removal of seven duplicated female records and correction of one male with inverted knee/hock measurements (subject M-7). Columns: ID = animal identifier; A = withers height; B = rump height; C = tail-insertion height; D = trunk length; E = head length; F = distance between auditory meatuses; G = distance between temporal angles of the eyes; CM = medial canthal distance; H = intermandibular distance; I = ear length; L = chest width; M = thoracic circumference; N = thoracic height; O = thoracic width; *p* = rump length; Q = anterior rump width; X = posterior rump width; R = sternum-to-ground distance; S = shoulder length; T = knee-to-ground distance; U = knee circumference; V = cannon (shin) circumference; W = hock-to-ground distance; Z = hock circumference; Weight (kg); BCS = Body Condition Score on a 1–5 donkey-specific scale; Sex = F (female) or M (male). All linear measurements are reported in cm; Table S3: Linear Discriminant Analysis (LDA) results for sex classification from the 25 morphometric traits in the Abruzzo donkey sample. The table reports (i) apparent classification accuracy, (ii) leave-one-out cross-validation (LOO-CV) accuracy, and (iii) the LOO-CV confusion matrix (rows = observed sex; columns = predicted sex). The LDA reaches 100% apparent accuracy and 93.7% cross-validated accuracy, with three females misclassified as males and one male misclassified as a female. Animals with missing BCS values were excluded from the LDA (effective *n* = 54 F + 9 M = 63); Table S4: Shapiro–Wilk normality test results for each of the 25 morphometric traits separately by sex. For each trait the table reports the Shapiro–Wilk *p*-value and a “Normal” flag (yes/no) based on the conventional α = 0.05 threshold. These results were used to decide, for each trait, whether to apply a Welch *t*-test (both groups normal) or a Mann–Whitney U test (at least one group non-normal) in the sexual dimorphism analysis (Table 3 of the main manuscript); Table S5: Zoometric indices computed for each Abruzzo donkey (*n* = 69), based on the morphometric traits of Table S2. BI, Body index (D/M × 100); TI, Thoracic index (L/N × 100); GS, Gracility shin index (V/A × 100); WHR, Weight–height ratio (Weight/A); CoI, Compactness index (Weight/D × 100); DTI, Dactyl-thoracic index (V/M × 100); CeI, Cephalic index (F/E × 100); Table S6: Two-way analysis of variance of each morphometric trait with Sex (fixed) and Farm (fixed) effects, on the *n* = 69 adult animals. The first column reports the *p*-value of the Sex effect estimated by the Welch *t*-test (univariate, used in Table 3 of the main manuscript); the second column reports the *p*-value of the Sex partial effect estimated by the two-way Type-III ANOVA after adjustment for Farm (car::Anova); the third column reports the intra-class correlation coefficient (ICC) per Farm computed on the female subset. All values are generated by the analysis_asino_abruzzese_v5.R script. The ‘Note’ column summarises the verdict per trait: ‘size’ = sex effect preserved after Farm adjustment; ‘size (mod. farm)’ = preserved but with non-negligible farm effect (ICC ≥ 0.20); ‘loses sig.’ = significant in Welch but not after Farm adjustment; ‘GAINS sig.’ = non-significant in Welch but significant after Farm adjustment (Farm was masking the sex effect); ‘ns’ = non-significant in both tests. Table S7: Quantification of the morphological harmony of the Abruzzo donkey following the classical zootechnical criterion of Bonadonna (1959), Aparicio Sanchez (1960) and Folch and Jordana (1997) [13,14,15]. The number and percentage of pair-wise Pearson correlations among the 25 linear morphometric traits (*n* = 300 total correlations) that are positive and BH-significant (q < 0.05) are reported, separately for the whole population, the female component and the male component. The 50% threshold for harmonicity is exceeded in both the whole population and the female subset. All values are reproducible from the analysis_asino_abruzzese_v5.R script (HARMONY block, between Figure 4 and Figure 5). Data and R code available on Zenodo (https://doi.org/10.5281/zenodo.20781399).
