# Supplementary material for: Phenotypic Characterisation of the Abruzzo Donkey (Equus asinus), an Endangered Italian Genetic Resource: Body Measurements
Source: Animals (Basel). 2026 Jun 22;16(12):1932. doi: 10.3390/ani16121932 (PMC13295899; doi:10.3390/ani16121932)
Supplement: Supplementary file 1 [file animals-16-01932-s001.zip › animals-4356570-supplementary.pdf]

Supplementary Materials

Phenotypic Characterization of the Abruzzo Donkey (*Equus asinus*), an Endangered Italian Genetic Resource: Body Measurements

Ippolito De Amicis 1, Vincenzo Landi 2,*, Alberto De Berardinis 1, Medhat S. Saleh 2,3, Ivano Massirio 1, Domenico Robbe 1, Roberta Bucci 1 and Augusto Carluccio 1

[1] Department of Veterinary Medicine, University of Teramo, Piano d'Accio, 64100 Teramo, Italy; ideamicis@unite.it (I.D.A.); alberto.deberardinis@unite.studenti.it (A.D.B.); imassirio@unite.it (I.M.); drobbe@unite.it (D.R.); acarluccio@unite.it (A.C.)

[2] Department of Veterinary Medicine, University of Bari Aldo Moro, S.P. 62 per Casamassima km. 3, Valenzano, 70010 Bari, Italy; medhat.elshahat@uniba.it

[3] Department of Animal Production, Faculty of Agriculture, Benha University, 13736 Benha, Egypt

[4] Department of Clinical Sciences and Translational Medicine, University Degli Studi Roma "Tor Vergata", Via Montpellier 1, 00133 Roma, Italy; rbuccivet@gmail.com (R.B.)

* Correspondence: vincenzo.landi@uniba.it

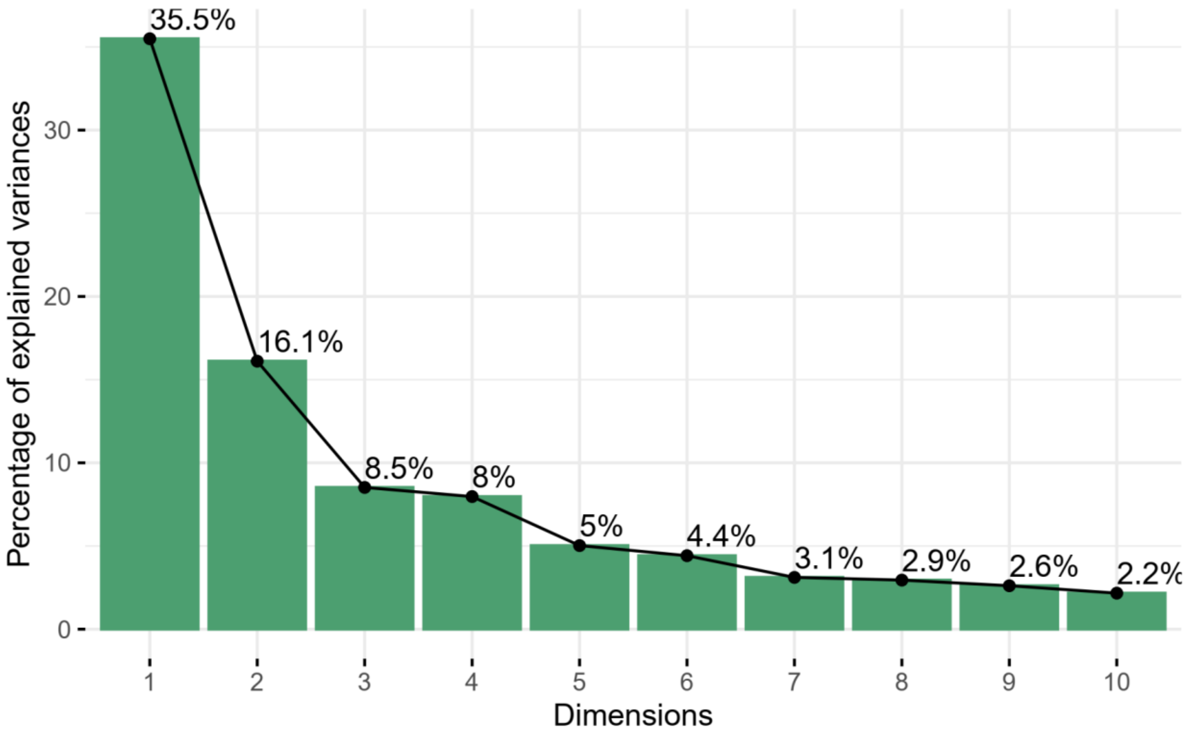

Figure S1. Principal component analysis (PCA): scree plot showing the percentage of total variance explained by each principal component (PC1 to PC25). PC1 captures the dominant size-related variation; PC2 captures the main proportional/conformation gradient. The 'elbow' criterion suggests that the first two components are sufficient to summarise the morphometric variability in the dataset.

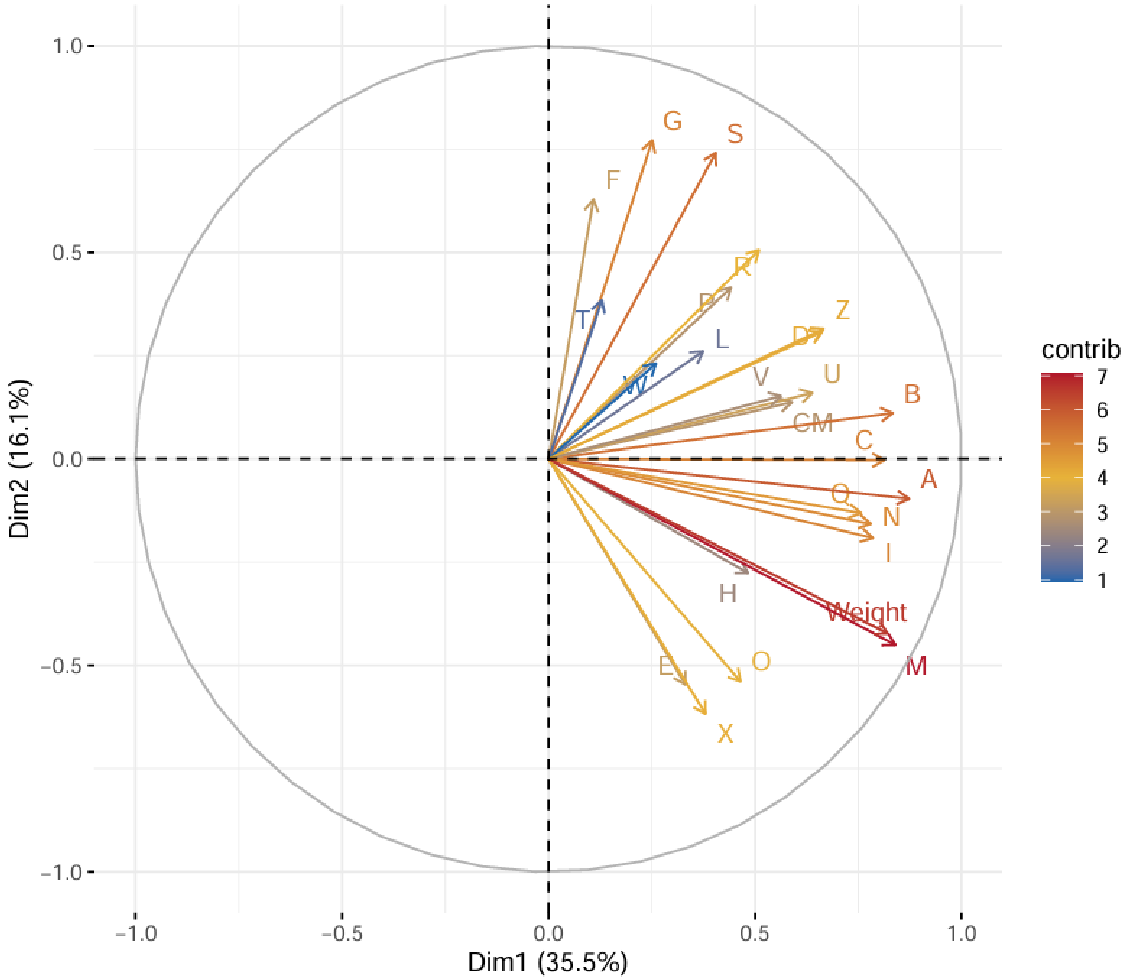

Figure S2. PCA variable contribution plot. Each arrow represents one of the 25 morphometric traits projected on the plane of the first two principal components (PC1 vs PC2). Arrow length reflects the trait's loading on the two axes and arrows are coloured by their relative contribution to the explained variance (cool to warm gradient). Variables pointing in the same direction are positively correlated; opposite directions indicate negative correlation

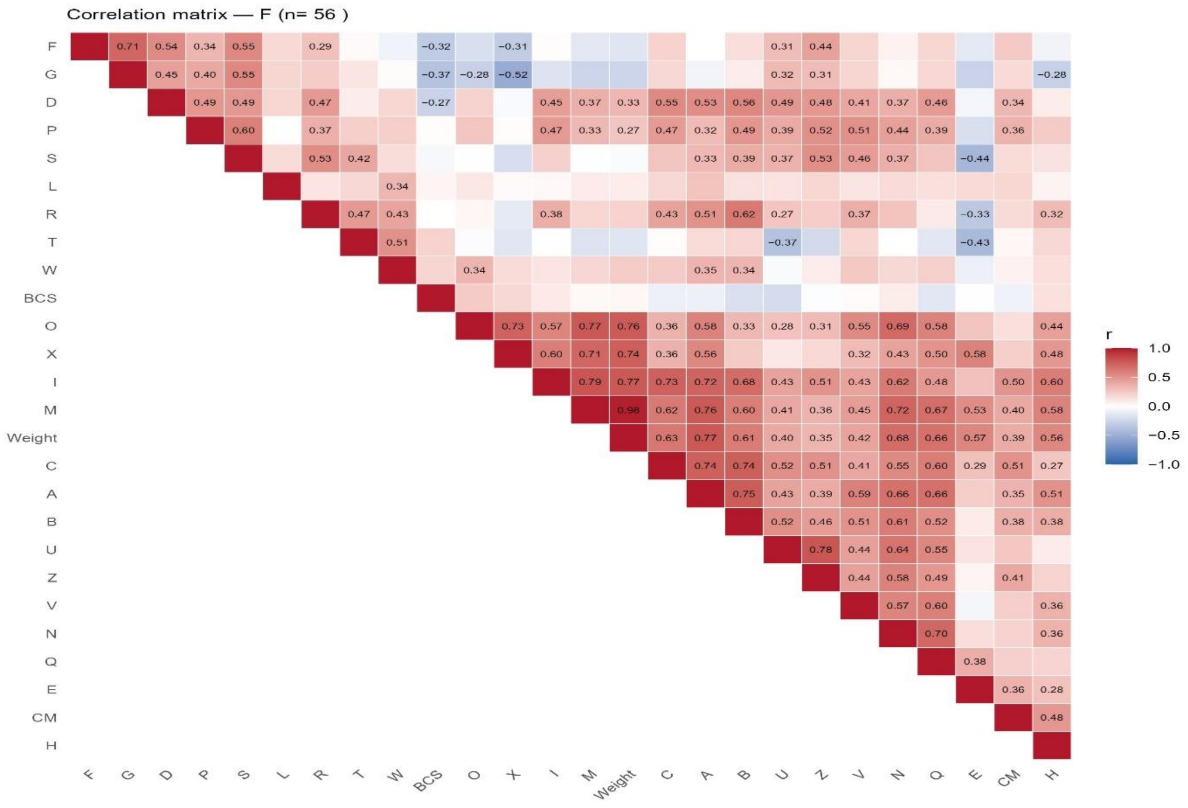

Figure S3. Pearson correlation heat-map of the 25 morphometric traits in female Abruzzo donkeys (n = 56). Only correlations statistically significant after Benjamini–Hochberg correction (q < 0.05) are displayed. Cell colour intensity reflects the magnitude and sign of the correlation coefficient (blue = positive, red = negative). Hierarchical clustering of traits was used to organise the matrix.

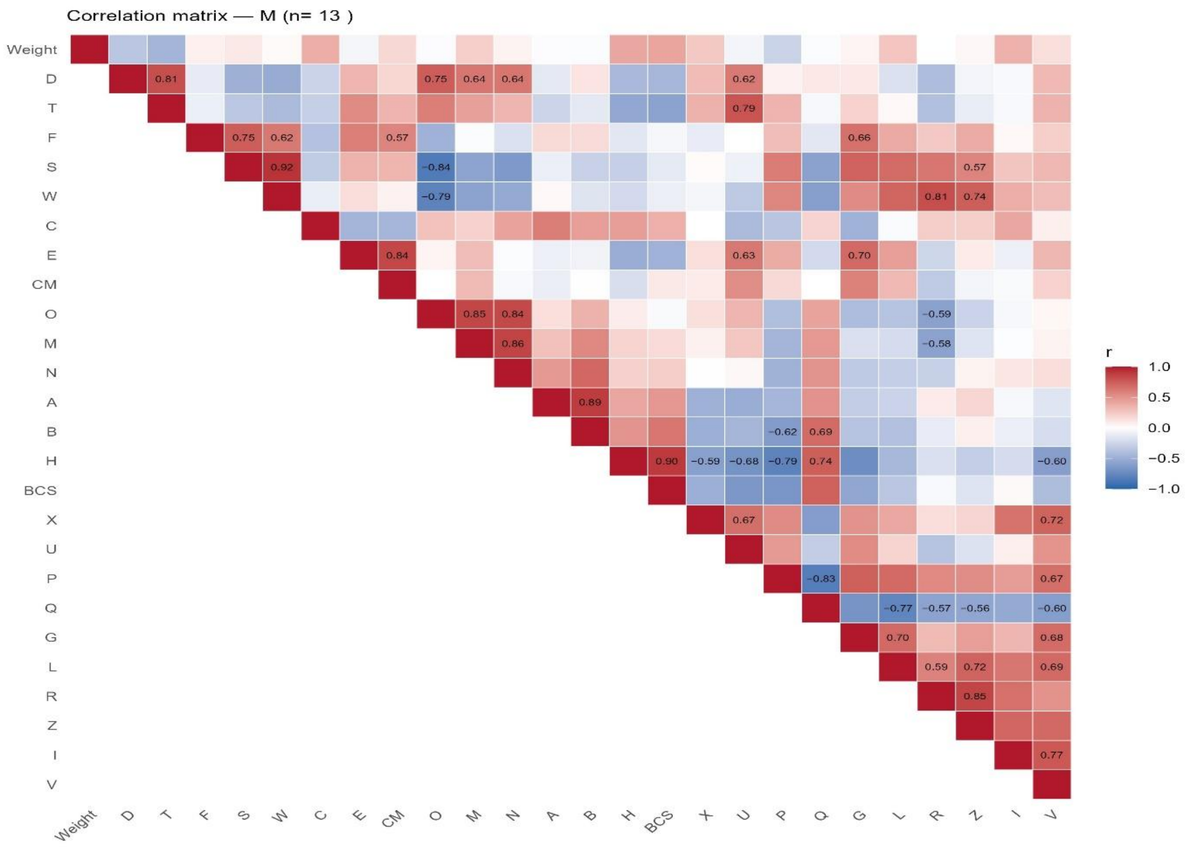

Figure S4. Pearson correlation heat-map of the 25 morphometric traits in male Abruzzo donkeys (n = 13). Display rules and clustering are as in Figure S3. The lower number of significant correlations compared to females reflects the smaller sample size of the male group and should not be interpreted as biological discordance.

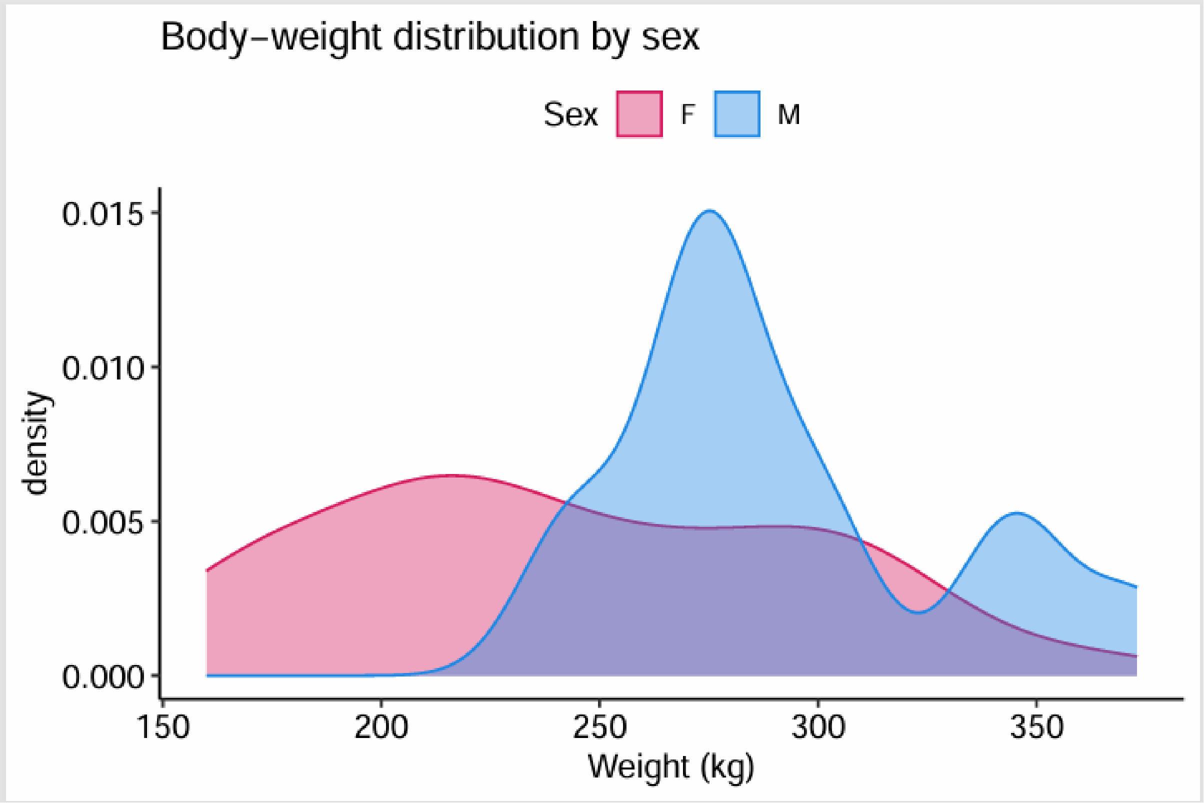

Figure S5. Kernel density plot of body weight (kg) distributions by sex in the Abruzzo donkey sample. Females (pink) and males (blue) are shown on the same scale, with each curve normalised to integrate to one. The horizontal shift highlights the sexual dimorphism in body mass (mean F = 242.5 kg; mean M = 292.4 kg).

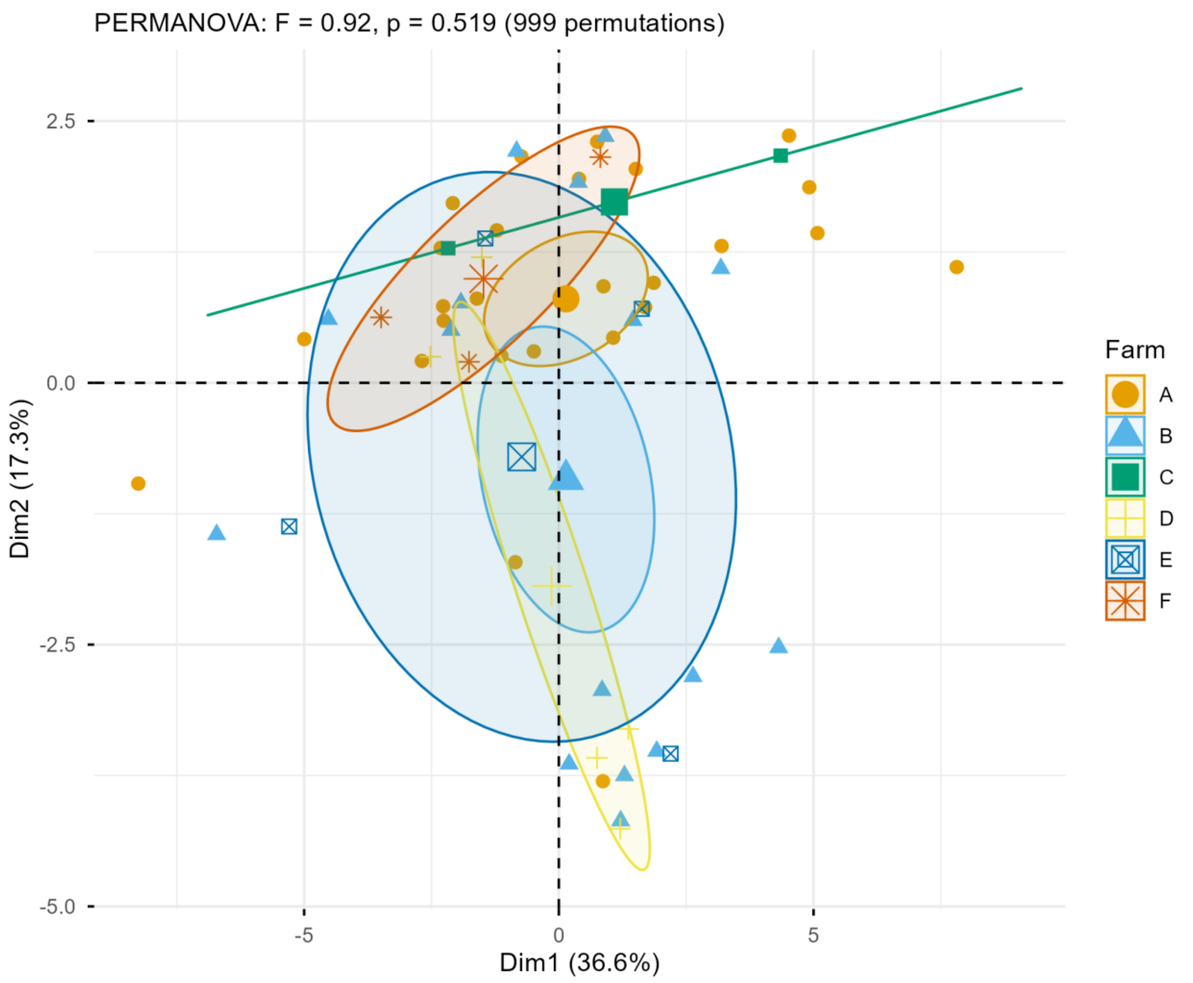

Figure S6. Principal Component Analysis (PCA) of the 22 linear morphometric traits in the 56 adult females of the Abruzzo donkey, colored by farm of origin (Farms A–F, see Table S5). Confidence ellipses (95%) of farms with n ≥ 3 individuals overlap entirely, indicating the absence of multivariate farm clustering (PERMANOVA F = 0.92, p = 0.519, 999 permutations; vegan::adonis2). This result, together with the negligible average intra-class correlation across single traits (mean ICC = 0.031; only the head width G, the coxal-tuber width X and the body condition score BCS — environmentally and ontogenetically labile traits — exceed ICC = 0.15), supports the morphological homogeneity of the breed across the surveyed farms. The PCA plot is produced by the analisi_asino_abruzzese_v5.R script.

Table S1. Distribution of the n = 69 Abruzzo donkeys among the six farms enrolled in the present study. Farms are anonymized (Farm A–F) to protect the privacy of the owners; only municipality and province are reported. All adult animals (≥ 3 years) present in each farm at the time of the survey were measured (exhaustive census-level sampling at farm level).

Farm	Municipality	Province	Females	Males	Total
A	Crognaleto	TE	26	2	28
B	Crognaleto	TE	16	5	21
C	Introdacqua	AQ	2	1	3
D	L'Aquila	AQ	5	2	7
E	Capestrano	AQ	4	2	6
F	Rocca Santa Maria	TE	3	1	4
TOTAL	—	—	56	13	69

Note: A and B are two adjacent farms in the same municipality, both managed under traditional pastoral conditions in the Crognaleto valley (Central Apennines). The remaining four farms are distributed across the Provinces of L'Aquila (C–E) and Teramo (F). The unbalanced farm distribution (Farms A + B = 71% of the sample) reflects the actual demography of the breed, which is heavily concentrated in two large extensive-grazing nuclei.

Table S2. Cleaned full dataset of Abruzzo donkey morphometric measurements (n = 69: 56 females + 13 males) used in the statistical analyses, after removal of seven duplicated female records and correction of one male with inverted knee/hock measurements (subject M-7). Columns: ID = animal identifier; A = withers height; B = rump height; C = tail-insertion height; D = trunk length; E = head length; F = distance between auditory meatuses; G = distance between temporal angles of the eyes; CM = medial canthal distance; H = intermandibular distance; I = ear length; L = chest width; M = thoracic circumference; N = thoracic height; O = thoracic width; P = rump length; Q = anterior rump width; X = posterior rump width; R = sternum-to-ground distance; S = shoulder length; T = knee-to-ground distance; U = knee circumference; V = cannon (shin) circumference; W = hock-to-ground distance; Z = hock circumference; Weight (kg); BCS = Body Condition Score on a 1–5 donkey-specific scale; Sex = F (female) or M (male). All linear measurements are reported in cm.

ID	A	B	C	D	E	F	G	CM	H	I	L	M	N	O	P	Q	X	R	S	T	U	V	W	Z	Weight	BCS	Sex
**1**	137	143	134	147	60	21	36	22	9	35	29	162	66	54	48	54	32	64	50	37	34	21	51	38	355	2	F
**2**	134	132	139	137	52	20	33	23	11	36	28	148	59	49	45	43	30	75	49	41	30	18	56	40	277	4	F
**3**	124	122	112	126	52	14	31	21	11	33	26	156	55	53	45	39	27	67	42	36	25	18	44	30	318	3.5	F
**4**	128	137	123	131	57	21	33	21	12	34	29	142	55	47	41	41	29	72	46	41	28	16	55	38	300	3	F
**5**	124	129	116	131	56	20	34	23	13	31	33	141	59	53	41	41	28	68	45	42	27	18	52	31	238	3	F
**6**	120	128	121	129	51	20	31	22	10	31	27	144	54	48	41	42	22	68	39	39	26	17	47	31	250	3	F
**7**	125	133	124	134	57	22	34	22	11	34	28	150	67	49	44	45	24	70	47	27	44	19	37	49	283	3	F
**8**	122	128	116	130	56	20	31	23	12	30	30	145	58	46	39	41	20	64	45	38	26	18	43	33	256	3	F
**9**	128	132	118	129	53	18	30	23	12	31	28	140	58	43	44	41	24	69	45	42	28	18	50	34	230	3	F
**10**	119	126	115	128	47	17	29	22	12	33	26	127	53	43	44	38	24	68	47	42	28	17	49	33	173	4	F
**11**	121	130	118	134	52	20	31	23	10	33	27	141	57	46	47	43	25	66	48	41	30	18	47	38	235	3	F
**12**	120	128	115	126	52	21	30	22	11	33	26	129	54	35	42	37	23	66	48	39	26	17	28	34	177	3	F
**13**	106	116	105	118	61	17	28	22	9	28	29	125	46	23	38	35	21	57	39	35	24	11	44	31	160	3	F
**14**	124	121	114	130	56	21	31	22	12	33	33	144	60	53	44	43	29	66	49	41	27	18.5	49	37	250	4	F
**15**	120	130	115	130	49	21	30	22	11	32	29	138	57	48	46	39	23	67	48	39	28	18	52	40	220	4	F
**16**	116	121	113	115	48	17	27	19	9	29	25	125	53	41	40	37	23	66	45	40	23	16	48	29	160	3.5	F
**17**	114	126	114	122	52	18	29	20	10	31	29	136	58	47	44	36	22	65	43	41	27	17	48	32	210	4	F
**18**	123	134	117	132	51	20	31	19	10	32	29	138	57	45	44	43	23	72	46	39	27	18	51	34	220	3	F
**19**	124	120	108	130	50	20	30	19	10	29	30	134	53	50	40	40	26	64	45	39	26	18	48	33	200	3	F
**20**	123	130	110	124	49	18	30	22	10	30	30	130	56	44	40	38	24	73	48	43	28	19	55	35	205	3	F
**21**	128	136	123	134	56	21	31	23	13	36	30	147	57	46	45	40	25	73	46	41	29	19	51	35	266	3	F
**22**	131	139	125	135	51	19	31	24	13	35	29	148	59	50	49	41	24	74	50	44	28	19	53	38	272	4	F
**23**	121	125	107	127	51	18	29	20	11	28	26	134	55	43	42	40	21	70	45	42	27	18	49	30	200	4	F
**24**	114	125	107	128	49	20	29	21	12	32	19	139	55	48	45	39	25	67	47	38	26	17	47	33	225	3	F
**25**	119	125	110	115	51	18	28	20	11	29	27	129	55	42	42	41	25	66	44	40	24	18	50	33	177	3.5	F

Cont. Table S2. Cleaned full dataset

ID	A	B	C	D	E	F	G	CM	H	I	L	M	N	O	P	Q	X	R	S	T	U	V	W	Z	Weight	BCS	Sex
**26**	128	130	118	135	56	16	18	22	12	36	27	154	57	54	38	39	33	70	40	38	26	17	49	33	306	4	F
**27**	137	141	126	126	66	19	25	24	13	34	33	164	59	52	43	43	34	74	44	39	28	17	55	32	367	3	F
**28**	130	135	122	138	60	16	24	20	10	34	27	155	65	60	42	44	32	63	43	39	29	17	49	36	312	4	F
**29**	122	126	118	121	65	19	26	23	13	34	24	151	57	55	43	43	37	63	40	38	26	19	47	35	289	3	F
**30**	130	133	116	120	58	15	20	19	14	34	27	157	61	48	40	41	27	71	46	40	27	17	47	32	324	4	F
**31**	121	125	117	118	64	18	25	22	12	33	26	150	56	54	42	42	36	63	42	37	26	18	47	34	311	5	F
**32**	128	131	115	118	55	14	20	21	13	34	29	151	58	56	43	41	37	62	43	38	27	19	49	35	290		F
**33**	127	130	116	117	56	15	25	22	12	35	27	155	65	60	42	44	32	63	43	39	29	17	49	36	312	4	F
**41**	105	117	105	117	61	17	28	21	9	28	29	125	46	43	39	37	21	57	39	35	25	15	44	31	160	4	F
**42**	123	121	114	130	56	20	31	22	12	32	32	144	60	53	44	43	29	66	49	41	27	18.5	49	37	250	5	F
**43**	121	131	115	131	49	21	30	22	11	32	30	138	57	48	46	39	23	67	48	39	28	18	52	40	220	4	F
**44**	117	122	113	116	48	18	27	20	9	30	27	125	53	41	40	37	23	66	45	40	23	16	48	29	160	5	F
**45**	115	125	114	121	52	18	29	20	10	31	29	136	58	47	44	38	22	65	43	41	27	17	48	32	210	4	F
**46**	122	133	117	133	51	19	31	19	10	32	29	138	57	45	44	43	23	72	46	40	28	18	51	34	220	4	F
**47**	125	121	108	132	50	20	30	19	10	30	30	134	53	50	40	40	26	64	45	39	27	18	48	33	200	3	F
**48**	122	131	110	125	49	18	30	21	10	30	30	130	56	44	40	38	24	73	48	43	28	19	55	35	205	4	F
**49**	127	135	123	134	56	20	31	23	13	37	30	147	57	46	45	40	25	73	46	41	29	19	51	35	266	4	F
**50**	130	138	125	134	51	18	31	24	13	35	29	148	59	50	49	41	24	74	50	44	28	19	53	38	272	4	F
**51**	120	126	107	128	51	18	29	20	11	29	26	134	55	43	42	40	21	70	45	42	27	18	49	30	200	4	F
**52**	115	124	107	128	49	21	29	21	12	31	24	139	55	48	45	39	25	67	47	39	26	17	47	33	225	3	F
**53**	118	125	110	116	51	18	28	20	11	30	29	129	55	42	42	41	25	66	44	40	24	18	50	33	177	4	F
**54**	129	131	118	134	56	17	18	22	12	36	29	154	57	54	42	39	33	70	40	38	26	17	49	33	306	4	F
**55**	122	124	118	118	64	18	25	22	12	33	27	151	57	55	42	43	36	63	42	38	27	19	47	34	310	5	F
**56**	127	130	116	118	55	14	20	21	13	34	29	152	57	56	42	42	37	62	43	38	28	19	49	35	290		F
**57**	128	130	116	117	56	15	25	22	12	35	28	154	66	61	42	43	33	63	43	39	29	18	49	36	312	4	F
**58**	121	129	115	126	52	21	30	22	11	33	26	134	55	37	42	38	25	66	48	39	27	17	30	34	190	3	F
**59**	116	117	114	120	61	17	28	22	10	28	29	126	47	38	39	37	22	58	39	36	25	16	44	31	165	3	F
**60**	125	122	114	130	56	21	31	22	12	33	33	145	61	52	44	43	29	67	49	41	27	17	49	37	250	4	F
**61**	118	129	115	130	49	21	30	22	11	32	29	140	58	50	45	40	23	67	48	40	28	18	52	40	220	4	F
**62**	118	122	113	115	48	17	27	19	10	29	29	128	54	46	42	38	23	68	45	41	27	16	48	31	179	3	F
**63**	115	127	114	122	52	18	29	20	10	31	29	139	59	48	44	37	25	66	43	40	27	17	48	32	225	4	F
**1**	127	133	121	126	56	19	32	26	14.5	33	32	148	55	35	42	43	24	70	50.5	39	27	16	53	36	345		M
**2**	130	135	117	133	55	19	33	25	12.5	31	29	145	56	29	42	45	20	72	50	39.5	27	16.5	51	36	243		M

Cont. Table S2.

ID	A	B	C	D	E	F	G	CM	H	I	L	M	N	O	P	Q	X	R	S	T	U	V	W	Z	Weight	BCS	Sex
**3**	133	139	123	147	57	18	32	27	13	33	30	153	64	57	39	46	24	66	43	41	29	17	44	36	300	5	M
**4**	130	135	128	137	53	15	27	22	14	32	29	148	60	52	40	45	22	70	40	40	26	16	44	35	272		M
**5**	129	134	127	135	54	17	33	25	13	36	31	148	59	44	42	43	33	73	47	39	30	19	47	38	373	5	M
**6**	132	134	126	138	58	18	35	26	9	34	33	148	58	45	45	41	29	72	49	42	32	19	51	40	272	3	M
**7**	124	132	116	147	57	16	31	27	12	30	28	150	58	57	41	46	27	60	40	43	35	16	35	27	283	4	M
**8**	125	133	117	148	58	19	36	27	10	35	34	149	59	46	46	42	30	73	50	43	33	20	52	41	273	4	M
**9**	123	131	115	146	56	17	34	25	9	33	32	147	57	44	44	40	28	71	48	41	31	18	50	39	271	3	M
**10**	129	134	122	127	57	18	33	27	14	32	33	147	56	36	41	44	19	70	51	39	28	17	52	37	344	5	M
**11**	131	135	118	134	56	18	32	26	12	32	30	146	55	37	43	44	20	71	50	40	28	16	52	36	245	5	M
**12**	134	138	122	147	57	19	31	26	14	31	29	152	63	56	40	47	19	67	45	41	29	17	45	37	302		M
**13**	131	136	127	138	54	16	28	25	15	34	30	149	61	53	41	46	21	71	43	41	27	17	45	38	278	5	M

Table S3. Linear Discriminant Analysis (LDA) results for sex classification from the 25 morphometric traits in the Abruzzo donkey sample. The table reports (i) apparent classification accuracy, (ii) leave-one-out cross-validation (LOO-CV) accuracy, and (iii) the LOO-CV confusion matrix (rows = observed sex; columns = predicted sex). The LDA reaches 100% apparent accuracy and 93.7% cross-validated accuracy, with three females misclassified as males and one male misclassified as a female. Animals with missing BCS values were excluded from the LDA (effective n = 54 F + 9 M = 63).

**Metric**	**Value**	**Confusion matrix (CV)**	
		Female	Male
**Apparent accuracy**	1	51	3
**Leave-one-out CV accuracy**	0.937	1	8

Table S4. Shapiro–Wilk normality test results for each of the 25 morphometric traits separately by sex. For each trait the table reports the Shapiro–Wilk p-value and a "Normal" flag (yes / no) based on the conventional $\alpha = 0.05$ threshold. These results were used to decide, for each trait, whether to apply a Welch t-test (both groups normal) or a Mann–Whitney U test (at least one group non-normal) in the sexual dimorphism analysis (Table 3 of the main manuscript).

Trait	Female		Male	
	**Shapiro_p**	**Normal**	**Shapiro_p**	**Normal**
**A**	0.3151	yes	0.5052	yes
**B**	0.7488	yes	0.6354	yes
**C**	0.0012	no	0.2263	yes
**D**	0.0166	no	0.1072	yes
**E**	0.0009	no	0.2119	yes
**F**	0.0019	no	0.0799	yes
**G**	0.0001	no	0.4208	yes
**CM**	0.0007	no	0.0076	no
**H**	0.002	no	0.1073	yes
**I**	0.0862	yes	0.9141	yes
**L**	0.0003	no	0.4176	yes
**M**	0.1433	yes	0.4411	yes
**N**	0.0011	no	0.4263	yes
**O**	0.019	no	0.349	yes
**P**	0.0795	yes	0.562	yes
**Q**	0	no	0.6056	yes
**X**	0	no	0.2287	yes
**R**	0.0779	yes	0.0056	no
**S**	0.0237	no	0.0481	no
**T**	0	no	0.1324	yes
**U**	0	no	0.303	yes
**V**	0	no	0.0302	no
**W**	0	no	0.0252	no
**Z**	0.0002	no	0.0078	no
**Weight**	0.0571	yes	0.1045	yes
**BCS**	0	no	0.0052	no

Table S5. Zoometric indices computed for each Abruzzo donkey (n = 69), based on the morphometric traits of Table S2. BI, Body index (D / M × 100); TI, Thoracic index (L / N × 100); GS, Gracility shin index (V / A × 100); WHR, Weight–height ratio (Weight / A); CoI, Compactness index (Weight / D × 100); DTI, Dactyl-thoracic index (V / M × 100); CeI, Cephalic index (F / E × 100).

ID	Sex	BI	TI	GS	WHR	CI	DTI	CI
**1**	F	90.74074	43.93939	15.32847	2.591241	241.4966	12.96296	35
**2**	F	92.56757	47.45763	13.43284	2.067164	202.1898	12.16216	38.46154
**3**	F	80.76923	47.27273	14.51613	2.564516	252.381	11.53846	26.92308
**4**	F	92.25352	52.72727	12.5	2.34375	229.0076	11.26761	36.84211
**5**	F	92.9078	55.9322	14.51613	1.919355	181.6794	12.76596	35.71429
**6**	F	89.58333	50	14.16667	2.083333	193.7984	11.80556	39.21569
**7**	F	89.33333	41.79104	15.2	2.264	211.194	12.66667	38.59649
**8**	F	89.65517	51.72414	14.7541	2.098361	196.9231	12.41379	35.71429
**9**	F	92.14286	48.27586	14.0625	1.796875	178.2946	12.85714	33.96226
**10**	F	100.7874	49.0566	14.28571	1.453782	135.1563	13.38583	36.17021
**11**	F	95.03546	47.36842	14.87603	1.942149	175.3731	12.76596	38.46154
**12**	F	97.67442	48.14815	14.16667	1.475	140.4762	13.17829	40.38462
**13**	F	94.4	63.04348	10.37736	1.509434	135.5932	8.8	27.86885
**14**	F	90.27778	55	14.91935	2.016129	192.3077	12.84722	37.5
**15**	F	94.2029	50.87719	15	1.833333	169.2308	13.04348	42.85714
**16**	F	92	47.16981	13.7931	1.37931	139.1304	12.8	35.41667
**17**	F	89.70588	50	14.91228	1.842105	172.1311	12.5	34.61538
**18**	F	95.65217	50.87719	14.63415	1.788618	166.6667	13.04348	39.21569
**19**	F	97.01493	56.60377	14.51613	1.612903	153.8462	13.43284	40
**20**	F	95.38462	53.57143	15.44715	1.666667	165.3226	14.61538	36.73469
**21**	F	91.15646	52.63158	14.84375	2.078125	198.5075	12.92517	37.5
**22**	F	91.21622	49.15254	14.50382	2.076336	201.4815	12.83784	37.2549
**23**	F	94.77612	47.27273	14.87603	1.652893	157.4803	13.43284	35.29412
**24**	F	92.08633	34.54545	14.91228	1.973684	175.7813	12.23022	40.81633
**25**	F	89.14729	49.09091	15.12605	1.487395	153.913	13.95349	35.29412
**26**	F	87.66234	47.36842	13.28125	2.390625	226.6667	11.03896	28.57143
**27**	F	76.82927	55.9322	12.40876	2.678832	291.2698	10.36585	28.78788
**28**	F	89.03226	41.53846	13.07692	2.4	226.087	10.96774	26.66667
**29**	F	80.13245	42.10526	15.57377	2.368852	238.843	12.58278	29.23077
**30**	F	76.43312	44.2623	13.07692	2.492308	270	10.82803	25.86207
**31**	F	78.66667	46.42857	14.87603	2.570248	263.5593	12	28.125
**32**	F	78.1457	50	14.84375	2.265625	245.7627	12.58278	25.45455
**33**	F	75.48387	41.53846	13.38583	2.456693	266.6667	10.96774	26.78571
**41**	F	93.6	63.04348	14.28571	1.52381	136.7521	12	27.86885
**42**	F	90.27778	53.33333	15.04065	2.03252	192.3077	12.84722	35.71429
**43**	F	94.92754	52.63158	14.87603	1.818182	167.9389	13.04348	42.85714
**44**	F	92.8	50.9434	13.67521	1.367521	137.931	12.8	37.5
**45**	F	88.97059	50	14.78261	1.826087	173.5537	12.5	34.61538
**46**	F	96.37681	50.87719	14.7541	1.803279	165.4135	13.04348	37.2549
**47**	F	98.50746	56.60377	14.4	1.6	151.5152	13.43284	40
**48**	F	96.15385	53.57143	15.57377	1.680328	164	14.61538	36.73469

Cont. Table S5.

ID	Sex	BI	TI	GS	WHR	CI	DTI	CI
**49**	F	91.15646	52.63158	14.96063	2.094488	198.5075	12.92517	35.71429
**50**	F	90.54054	49.15254	14.61538	2.092308	202.9851	12.83784	35.29412
**51**	F	95.52239	47.27273	15	1.666667	156.25	13.43284	35.29412
**52**	F	92.08633	43.63636	14.78261	1.956522	175.7813	12.23022	42.85714
**53**	F	89.92248	52.72727	15.25424	1.5	152.5862	13.95349	35.29412
**54**	F	87.01299	50.87719	13.17829	2.372093	228.3582	11.03896	30.35714
**55**	F	78.1457	47.36842	15.57377	2.540984	262.7119	12.58278	28.125
**56**	F	77.63158	50.87719	14.96063	2.283465	245.7627	12.5	25.45455
**57**	F	75.97403	42.42424	14.0625	2.4375	266.6667	11.68831	26.78571
**58**	F	94.02985	47.27273	14.04959	1.570248	150.7937	12.68657	40.38462
**59**	F	95.2381	61.70213	13.7931	1.422414	137.5	12.69841	27.86885
**60**	F	89.65517	54.09836	13.6	2	192.3077	11.72414	37.5
**61**	F	92.85714	50	15.25424	1.864407	169.2308	12.85714	42.85714
**62**	F	89.84375	53.7037	13.55932	1.516949	155.6522	12.5	35.41667
**63**	F	87.76978	49.15254	14.78261	1.956522	184.4262	12.23022	34.61538
**1**	M	85.13514	58.18182	12.59843	2.716535	273.8095	10.81081	33.92857
**2**	M	91.72414	51.78571	12.69231	1.869231	182.7068	11.37931	34.54545
**3**	M	96.07843	46.875	12.78195	2.255639	204.0816	11.11111	31.57895
**4**	M	92.56757	48.33333	12.30769	2.092308	198.5401	10.81081	28.30189
**5**	M	91.21622	52.54237	14.72868	2.891473	276.2963	12.83784	31.48148
**6**	M	93.24324	56.89655	14.39394	2.060606	197.1014	12.83784	31.03448
**7**	M	98	48.27586	12.90323	2.282258	192.517	10.66667	28.07018
**8**	M	99.32886	57.62712	16	2.184	184.4595	13.42282	32.75862
**9**	M	99.31973	56.14035	14.63415	2.203252	185.6164	12.2449	30.35714
**10**	M	86.39456	58.92857	13.17829	2.666667	270.8661	11.56463	31.57895
**11**	M	91.78082	54.54545	12.21374	1.870229	182.8358	10.9589	32.14286
**12**	M	96.71053	46.03175	12.68657	2.253731	205.4422	11.18421	33.33333
**13**	M	92.61745	49.18033	12.9771	2.122137	201.4493	11.4094	29.62963

BI, Body index; TI, Thoracic index; GS, Gracility shin index; WHR, Weight–height ratio; CoI, Compactness index; DTI, Dactyl-thoracic index; CeI, Cephalic index.

Table S6. Two-way analysis of variance of each morphometric trait with Sex (fixed) and Farm (fixed) effects, on the n = 69 adult animals. The first column reports the p-value of the Sex effect estimated by the Welch t-test (univariate, used in Table 3 of the main manuscript); the second column reports the p-value of the Sex partial effect estimated by the two-way Type-III ANOVA after adjustment for Farm (car::Anova); the third column reports the intra-class correlation coefficient (ICC) per Farm computed on the female subset. All values are generated by the analyysis_asino_abruzzese_v5.R script. The 'Note' column summarises the verdict per trait: 'size' = sex effect preserved after Farm adjustment; 'size (mod. farm)' = preserved but with non-negligible farm effect (ICC ≥ 0.20); 'loses sig.' = significant in Welch but not after Farm adjustment; 'GAINS sig.' = non-significant in Welch but significant after Farm adjustment (Farm was masking the sex effect); 'ns' = non-significant in both tests.

Trait	p (Welch)	p (Sex ∣ Farm)	ICC_farm	Note
**A**	<1e-04	0.0014	0.000	size
**B**	<1e-06	0.0004	0.000	size
**C**	0.0012	0.0036	0.000	size
**D**	<1e-04	<1e-06	0.074	size
**E**	0.0107	0.279	0.085	loses sig.
**F**	0.0511	0.317	0.000	ns
**G**	0.0003	0.0001	0.226	size (mod. farm)
**CM**	<1e-06	<1e-06	0.000	size
**H**	0.0507	0.0151	0.000	GAINS sig.
**I**	0.322	0.512	0.000	ns
**L**	0.0005	0.0033	0.000	size
**M**	<1e-04	0.0376	0.000	size
**N**	0.103	0.272	0.000	ns
**O**	0.383	0.103	0.000	ns
**P**	0.237	0.329	0.000	ns
**Q**	<1e-04	0.0003	0.000	size
**X**	0.145	0.0467	0.157	GAINS sig.
**R**	0.0320	0.0174	0.065	size
**S**	0.191	0.0626	0.000	ns
**T**	0.0274	0.155	0.000	loses sig.
**U**	0.0263	0.0251	0.000	size
**V**	0.401	0.364	0.000	ns
**W**	0.729	0.805	0.020	ns
**Z**	0.0460	0.0457	0.000	size
**Weight**	0.0009	0.0052	0.000	size
**BCS**	0.0409	0.0795	0.187	loses sig.

Table S7. Quantification of the morphological harmony of the Abruzzo donkey following the classical zootechnical criterion of Bonadonna (1959), Aparicio Sanchez (1960) and Folch and Jordana (1997). The number and percentage of pair-wise Pearson correlations among the 25 linear morphometric traits ($n = 300$ total correlations) that are positive and BH-significant ($q < 0.05$) are reported, separately for the whole population, the female component and the male component. The 50% threshold for harmonicity is exceeded in both the whole population and the female subset. All values are reproducible from the analyysis_asino_abruzzese_v5.R script (HARMONY block, between Figure 4 and Figure 5).

Group	N	Positive sig.	%	Negative sig.	%	Verdict
**Whole population**	69	159 / 300	53.0	5 / 300	1.7	HARMONIC
**Females**	56	156 / 300	52.0	6 / 300	2.0	HARMONIC
**Males**	13	11 / 300	3.7	5 / 300	1.7	NOT harmonic*

*Note: the value of 3.7% in males reflects the limited statistical power of the male sub-sample ($n = 13$) under BH-FDR correction and not biological discordance — see Limitations section of the Discussion.
